# LATS-YAP/TAZ controls lineage specification by regulating TGFβ signaling and *Hnf4α* expression during liver development

**DOI:** 10.1038/ncomms11961

**Published:** 2016-06-30

**Authors:** Da-Hye Lee, Jae Oh Park, Tae-Shin Kim, Sang-Kyum Kim, Tack-hoon Kim, Min-chul Kim, Gun Soo Park, Jeong-Hwan Kim, Shinji Kuninaka, Eric N. Olson, Hideyuki Saya, Seon-Young Kim, Ho Lee, Dae-Sik Lim

**Affiliations:** 1 National Creative Research Initiatives Center for Cell Division and Differentiation, Department of Biological Science, Korea Advanced Institute of Science and Technology, Daejeon 34141, Republic of Korea; 2 Department of Pathology, Yonsei University College of Medicine, Seoul 03722, Republic of Korea; 3 Medical Genomics Research Center, Korea Research Institute of Bioscience and Biotechnology, 125 Gwahak-ro, Yuseong-gu, Daejeon 34141, Republic of Korea; 4 Division of Gene Regulation, Institute for Advanced Medical Research, Keio University School of Medicine, Tokyo 160-8582, Japan; 5 Department of Molecular Biology, University of Texas Southwestern Medical Center at Dallas, 5323 Harry Hines Boulevard, Dallas, Texas 75390–9148, USA; 6 National Cancer Center, Graduate School of Cancer Science and Policy, Research Institute, Goyang 10408, Republic of Korea

## Abstract

The Hippo pathway regulates the self-renewal and differentiation of various adult stem cells, but its role in cell fate determination and differentiation during liver development remains unclear. Here we report that the Hippo pathway controls liver cell lineage specification and proliferation separately from Notch signalling, using mice and primary hepatoblasts with liver-specific knockout of *Lats1* and *Lats2* kinase, the direct upstream regulators of YAP and TAZ. During and after liver development, the activation of YAP/TAZ induced by loss of *Lats1/2* forces hepatoblasts or hepatocytes to commit to the biliary epithelial cell (BEC) lineage. It increases BEC and fibroblast proliferation by up-regulating TGFβ signalling, but suppresses hepatoblast to hepatocyte differentiation by repressing *Hnf4α* expression. Notably, oncogenic YAP/TAZ activation in hepatocytes induces massive p53-dependent cell senescence/death. Together, our results reveal that YAP/TAZ activity levels govern liver cell differentiation and proliferation in a context-dependent manner.

## Abstract (web summary)

The Hippo pathway regulates the differentiation of stem and progenitor cells, but it is unclear how it acts in liver development. Here, the authors knockout Hippo pathway components *Lats1* and *2* in the liver, causing suppression of hepatocyte differentiation but promoting biliary cell differentiation.

## Graphical abstract

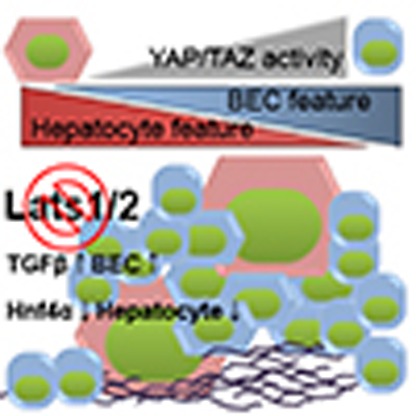

For proper organ development and maintenance, the self-renewal and differentiation of stem/progenitor cells must be spatiotemporally regulated. Throughout the development of an embryo, environmental cues trigger transcriptional changes in progenitor cells, which determine lineage specificities by multiple events^1^,^2^. In the embryonic liver, progenitor cells called hepatoblasts have the potential to differentiate into hepatocytes or intrahepatic biliary epithelial cells (BECs)^3^. These differentiation fates can be determined by the localization of the hepatoblast with respect to the portal vein^4^: hepatoblasts exposed to ligands from portal venous endothelial cells (for example, JAG1 and TGFβ) differentiate into primitive ductal plate cells and subsequently form bile ducts^5^, while hepatoblasts located further from the portal vein differentiate into hepatocytes^6^. During the neonatal period, hepatocytes proliferate rapidly to expand the liver mass while bile duct morphogenesis is completed. With respect to the control of hepatoblast differentiation, NOTCH, SOX9, HHEX, HNF6, ONECUT-2 and FOXM1B are known to specify BECs, while HNF1α, HNF1β, FOXA2, HNF4α, HNF6 and LRH-1 specify hepatocytes^4^. Adult hepatocytes, which are normally quiescent, proliferate in response to liver injury to recover the parenchyma. However, if hepatocytes are not able to restore more severely damaged livers, regeneration of liver requires the proliferation and differentiation of adult liver stem/progenitor cells through many signalling pathways and transcription factors^7^. It has been suggested that dysregulation of the involved factors can impair regeneration and trigger the development of cancer^8^.

The Hippo pathway, which restricts the proliferation of stem/progenitor cells and plays key roles in organ size control and regeneration^9^,^10^,^11^,^12^,^13^, comprises large tumour suppressors 1 and 2 (LATS1/2); MOB kinase activator 1A and 1B; the STE20-like kinases (MST1 and -2); neurofibromatosis 2 (NF2) and SAV1. On activation by upstream signals, MST1/2 phosphorylate and thereby activate LATS1/2 with the help of SAV1, and LATS1/2 then phosphorylate and inhibit the oncogenic transcriptional co-activators, YAP and TAZ (refs 14, 15). Many previous studies of the Hippo pathway have focused on adult liver carcinogenesis^16^,^17^,^18^. Such work has shown that *Nf2*-, *Mst1/2*- and *Sav1*-liver-specific knockout mice and YAP wild-type (WT) or S127A over-expressing mice exhibited varying degrees of hepatomegaly, over-proliferation of biliary/progenitor cells and hepatocellular carcinoma (HCC)^9^,^19^,^20^,^21^,^22^,^23^,^24^,^25^,^26^. In contrast, little research has focused on whether and how the Hippo pathway controls embryonic liver development.

Importantly, a recent study suggested that ectopic activation of YAP can convert mature hepatocytes into cells with hepatic progenitor properties in the adult liver, and that Notch signalling functions as a downstream target of YAP in this de-differentiation process^27^. However, the other targets of YAP responsible for controlling cell fate determination and the properties of such cells are unknown.

Here, using mice with liver-specific conditional knockout of both *Lats1/2*, we show that hyper-activation of YAP/TAZ, enhancement of BEC differentiation, and inhibition of hepatocyte differentiation *in vitro* and *in vivo*. Surprisingly, Notch signalling does not play an important role in the abnormal differentiation of *Lats1/2*-knockout hepatoblasts. Finally, we newly discover TGFβ signalling, HNF4α and the p53 pathway as downstream effectors of YAP/TAZ activation in the cell fate determination and differentiation of *Lats1/2*-deleted liver cells.

## Results

### *Lats1/2*-deleted hepatoblasts differentiate into BECs

**Figure 1:**
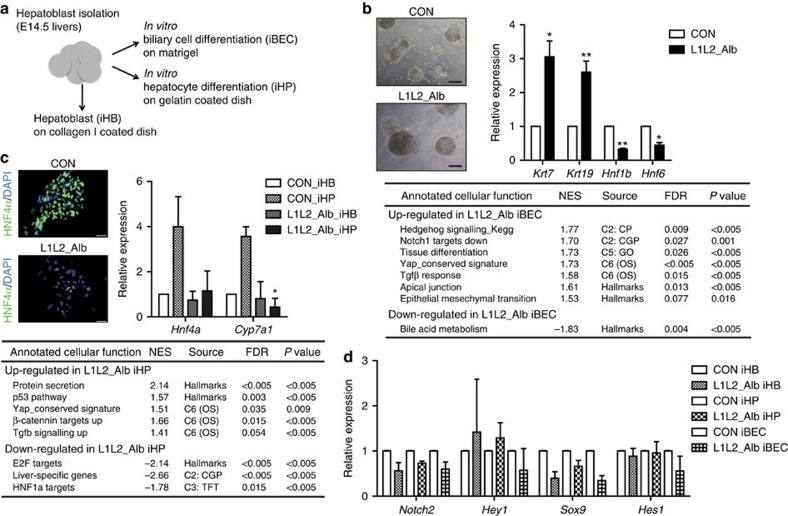
*Lats1/2*-deficient hepatoblasts strongly differentiate into BECs but not hepatocytes. (**a**) *In vitro* culture system schematic. (**b**) Representative bright-field images of BECs obtained from control and L1L2_Alb hepatoblasts subjected to *in vitro* differentiation for 14 days (upper). Scale bars, 400 μm. The graph shows the mRNA expression levels of *cytokeratins 7* and *19* in control and L1L2_Alb iBECs, as assessed by qRT-PCR. The box shows up-regulated or down-regulated gene sets from the RNA-sequencing results with control and L1L2_Alb iBECs. (**c**) Representative immunofluorescence staining with anti-HNF4α (left) and relative mRNA expression levels of *Hnf4α* and *Cyp7a1* in control and L1L2_Alb iHBs and iHPs, as assessed by qRT-PCR (right). Scale bars, 50 μm. The box shows up-regulated or down-regulated gene sets from the RNA-sequencing results with control and L1L2_Alb iHPs. (**d**) Relative expression levels of Notch signalling target genes in control and *Lats1/2*-deficient iHBs, iHPs and iBECs. The data are presented as means±s.e.m.; *n*=3 (**b**,**c**), *P<0.05 and **P<0.01 (Student's *t*-test).

The LATS1/2 kinases directly phosphorylate and inhibit the activities of YAP and TAZ in the Hippo pathway. To investigate the roles of LATS1/2 in liver cell specification, we generated a liver-specific *Lats1/2*-knockout mouse using the Albumin-Cre mouse system (*Lats1*^−/−^*; Lats2*^*fl/fl*^*; Alb-cre*; hereafter designated L1L2_Alb). We first isolated hepatoblasts from control and L1L2_Alb E14.5 livers, cultured them and examined their differentiation into either BECs or hepatocytes (Fig. 1a). We observed that a >50% reduction of LATS1/2 expression in the L1L2_Alb cells as well as a corresponding reduction in YAP. *Lats1/2*-deleted cells also showed an up-regulation of YAP target genes (Supplementary Fig. 1a–c). Compared with control hepatoblasts, *Lats1/2*-deficient hepatoblasts gave rise to more and larger BEC colonies, and the iBECs (*in vitro* differentiated BECs) were found to express more *cytokeratin 7* and *19*, which are BEC markers (Fig. 1b). For hepatocyte differentiation, we treated our *in vitro* culture system with the specific ligand, Oncostatin M. The transcription factor, HNF4α, which is critical for hepatocyte specification, was expressed normally in control iHPs (*in vitro* differentiated hepatocytes). Surprisingly, however, the expression levels of *Hnf4α, Cyp7a1*, which is an HNF4α target gene and a marker of mature hepatocytes^28^, along with many other hepatocyte-related genes were significantly reduced in *Lats1/2*-deficient iHPs (Fig. 1c and Supplementary Fig. 1d), suggesting that such cells are defective in their differentiation to hepatocytes. To investigate the signals downstream of LATS1/2-YAP/TAZ that guide hepatoblast lineage specification to hepatocytes and BECs, we performed an RNA-sequencing analysis with iHBs (*in vitro* cultured heptoblasts), iBECs and iHPs. Using gene set enrichment analysis, we found that along with the up-regulation of YAP target genes, *Lats1/2*-deficient iBECs and iHPs also show specific enrichment in several other signalling pathways (Fig. 1b,c and Supplementary Fig. 1c). JAG1-NOTCH-SOX9 signalling is known to trigger hepatoblast to BEC differentiation during liver development as well as hepatocyte de-differentiation^29^,^30^,^31^,^32^. Moreover, in the liver, mutations that trigger dysfunctions of NOTCH or JAG1 cause an absence of bile ducts similar to that seen in *Yap* deficient^33^,^34^,^35^. Notch signalling has been suggested as a target of YAP with regard to adult liver cell de-differentiation^27^. Thus, we expected an up-regulation of Notch signalling in *Lats1/2*-deficient cells. Instead, we found no significant change in Notch signalling in *Lats1/2*-deficient cells and an unexpected up-regulation of genes normally down-regulated by NOTCH1 activation in *Lats1/2*-deficient iBECs (Fig. 1b). Although we observed a significant increase in the mRNA expression level of *Jagged1* in *Lats1/2*-deficient cells, no such change was observed in the mRNA levels of *Notch2* and its target genes, *Sox9*, *Hes1* and *Hey1* (Fig. 1d). Thus, we hypothesized that some other signalling pathway, distinct from Notch, must be involved in the enhancement of BEC differentiation induced by loss of *Lats1/2* kinase.

### *Lats1/2*-deleted livers is distinct from N1ICD Tg livers

**Figure 2:**
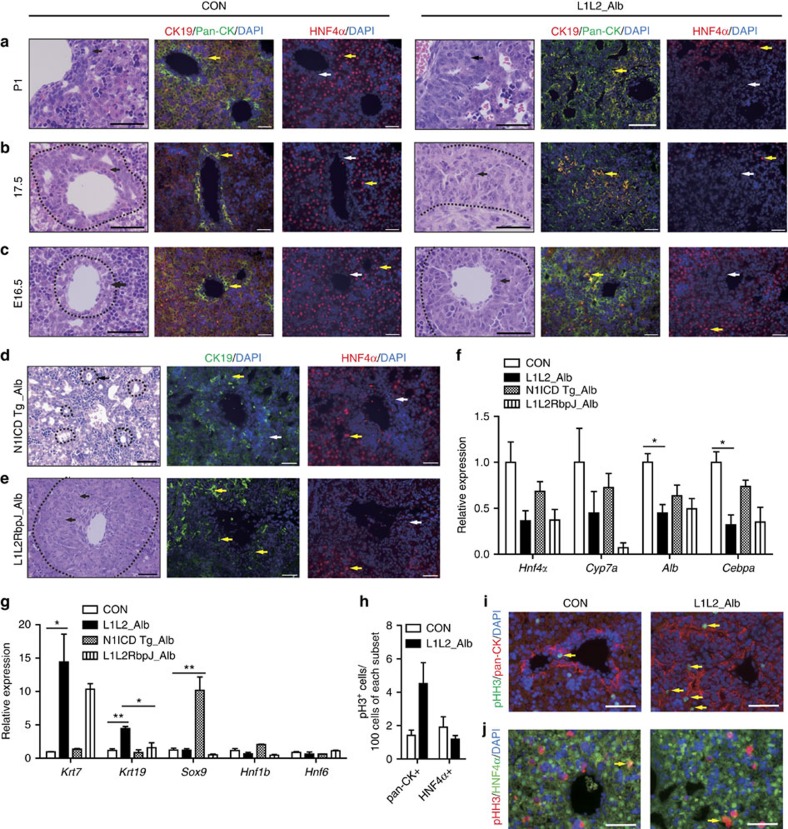
Loss of *Lats1* and *Lats2* in embryonic livers causes abnormal liver development. (**a**–**c**) Haematoxylin and eosin (H&E) staining (first and fourth column), immunofluorescence staining with anti-CK19, anti-pan-CK and diamidino-2-phenylindole (DAPI; second and fifth column), and anti-HNF4α and DAPI (third and sixth column) of control and L1L2_Alb livers at postnatal day (P) 1 (**a**), embryonic day (E) E17.5 (**b**) and E16.5 (**c**). Black arrows indicate ductal plate cells, and the dotted lines indicate ductal plates. Yellow arrows indicate positive antibody signal; white arrows indicate a lack of staining. Scale bars, 50 μm. (**d**,**e**) Representative H&E (left), immunofluorescence staining with anti-CK19 and DAPI (middle) and anti-HNF4α and DAPI (right) result of N1ICD Tg_Alb livers (**d**) and L1L2RbpJ_Alb livers (**e**) at P1. Black arrows indicate ductal plate cells, and dotted lines indicate bile ducts. Yellow arrows indicate positive antibody signal; white arrows indicate a lack of staining. Scale bars, 50 μm. (**f**,**g**) Relative mRNA expression levels of hepatocyte-related genes (**f**) and BEC-related genes (**g**) obtained from P1 livers of the indicated genotypes, as assessed by qRT-PCR. (**h**) The number of pHH3 (S10)-positive cells out of 100 pan-CK- and HNF4α-expressing cells in P1 control and L1L2_Alb livers. (**i**,**j**) Representative immunohistochemical staining results obtained using antibodies against phospho-Histone H3 (S10) and –pan-CK (**i**) or -HNF4α (**j**) of control and L1L2_alb livers at P1. Yellow arrows indicate double positive signal. Scale bars, 100 μm. The data are presented as means±s.e.m.; *n*=3, *P<0.05 and **P<0.01 (Student's *t*-test).

To demonstrate the *in vivo* role of LATS1/2 during liver development, we next analysed control and L1L2_Alb livers over the course of embryonic development (E). L1L2_Alb livers exhibited abnormal multilayered ductal plates at E16.5, when the ductal plate is forming and ductal cells rapidly proliferate. This ductal plate expansion grew more evident over developmental time as the *Lats1/2* levels dropped even further (Fig. 2a–c and Supplementary Fig. 2a). On postnatal period (P) P1, L1L2_Alb livers were filled with immature BECs and fibroblasts at the expense of functionally mature hepatocytes. Moreover, we found high levels of proliferation only among committed BECs, not committed hepatocytes (Fig. 2h–j). Finally, we found that the L1L2_Alb mice died before weaning, due to the large number of immature BECs and lack of functional hepatocytes (Fig. 2a and Supplementary Fig. 2b–d). It is noted that the size of L1L2_Alb liver was similar to control livers. The premature death of L1L2_Alb mice may not allow sufficient time for sufficient growth in the mutant livers.

Livers tissue-specifically over-expressing the Notch1 intracellular domain (N1ICD Tg_Alb) had more tubular intrahepatic bile ducts (Supplementary Fig. 3a) and ultimately develop cholangiocarcinomas. In contrast, L1L2_Alb livers, although they too contained few functional hepatocytes, consisted mainly of immature BECs that had not completed morphogenesis (Fig. 2a,d). Remarkably, the immature BECs in *Lats1/2*-deleted livers were strongly CK19-positive when compared with those found in N1ICD Tg_Alb livers (Fig. 2a,d). Microarray and quantitative reverse transcription-PCR (qRT-PCR) analyses of L1L2_Alb livers confirmed the up-regulation of BEC signature genes (*Krt7* and *Krt19*) and the down-regulation of hepatocyte signature genes (*Hnf4α*, *Cyp7a1* and *Cebpα*; Supplementary Table 1); the changes in the expression of these signature genes were more severe in L1L2_Alb livers than in N1ICD Tg_Alb livers (Fig. 2f,g). In addition, N1ICD overexpression was associated with high expression of bile duct morphogenesis-related genes, whereas we observed no such change in L1L2_Alb livers (Fig. 2g). Thus, it seems that, when compared with loss of *Lats1/2*, activation of Notch signalling induces a different, less severe reduction in embryonic liver function.

To further investigate the genetic relationship between the Notch and Hippo pathways, we generated *Lats1*^−/−^*; Lats2*^*fl/fl*^*; RbpJ*^*fl/fl*^*; Alb-cre* (L1L2RbpJ_Alb) mice and N1ICD *Tg; Yap*^*fl/fl*^*; Alb-cre* (N1ICD Tg; Yap_Alb) mice. The livers with a deletion of RbpJ (recombination signal binding protein for immunoglobulin kappa J region), a downstream effector of Notch signalling, showed complete absence of NOTCH1/2 activation and defective bile duct morphogenesis^36^. Still, L1L2RbpJ_Alb livers showed severe expansion of immature BECs although there was marginal reduction of BECs and increase of hepatocytes compared with L1L2_Alb livers (Fig. 2e–g). Moreover, *Yap* deficiency in N1ICD over-expressing mice significantly diminished cytokeratin expression without alteration in *Sox9* expression (Supplementary Fig. 3b–d). Based on these data, we speculated that, rather than acting downstream of YAP/TAZ activation, the Notch and Hippo pathways run in parallel. Thus, NOTCH-SOX9 signalling and Hippo-YAP/TAZ signalling are both important for lineage specification during liver development.

### The degree of BEC proliferation is tuned by YAP/TAZ activity

**Figure 3:**
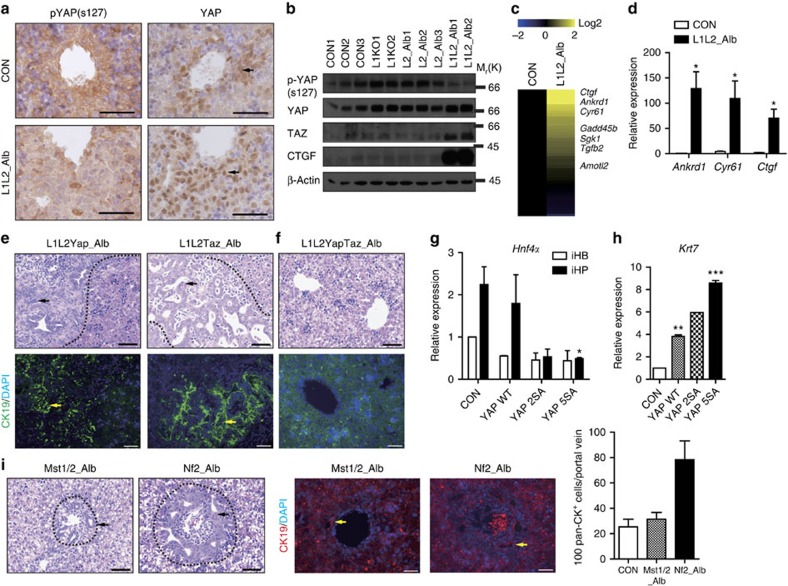
Proper activity of YAP and TAZ are essential for ductal plate expansion. (**a**) Representative immunohistochemical staining results obtained with anti-phospho-YAP (S127) and anti-YAP in control and L1L2_Alb livers sampled at E16.5. Black arrows indicate positive signals. (**b**) Western blot analysis of whole livers obtained at E17.5 from embryos of the indicated genotypes. (**c**,**d**) Heat map (**c**) and qRT-PCR (**d**) of YAP target genes, reflecting microarray data obtained with mRNAs from control and L1L2_Alb P1 livers. (**e**,**f**) Representative images of haematoxylin and eosin (H&E) staining (upper) and immunofluorescence staining (lower) with anti-CK19 and diamidino-2-phenylindole (DAPI) of *Lats1^−/−^; Lats2^fl/fl^; Yap^fl/fl^; Alb-Cre* (L1L2Yap_Alb), *Lats1^−/−^; Lats2^fl/fl^; Taz^fl/fl^; Alb-Cre* (L1LTaz_Alb) (**e**) and *Lats1^−/−^; Lats2^fl/fl^; Yap^fl/fl^; Taz^fl/fl^; Alb-Cre* (L1L2YapTaz_Alb) (**f**) livers at P1. Black dotted lines indicate boundary between ductal plates and non-ductal plates. Black arrows indicate ductal plate cells. Yellow arrows indicate CK19-positive signal. (**g**) Relative mRNA expression levels of *Hnf4α* in control, YAP WT, -2SA and -5SA over-expressing iHPs. (**h**) Quantification of *cytokeratin 7* mRNA expression in control, YAP WT, -2SA and -5SA over-expressing iBECs. *n*=2. (**i**) H&E staining (left) and immunofluorescene staining (right) with anti-CK19 and DAPI of *Mst1* and *-2* double-knockout livers and *Nf2* knockout livers at P1. The graph shows quantification of pan-CK-positive cells per portal triad. Black arrows indicate ductal plate cells, and the dotted lines indicate ductal plates. Yellow arrows indicate positive signals to CK19. Scale bars, 50 μm; the data are presented as means±s.e.m.; *n*=3, *P<0.05 and **P<0.01 (Student's *t*-test).

Since LATS1/2 directly phosphorylate YAP/TAZ and inhibit their activity, we next examined the localizations of YAP and phosphorylated YAP (S127). In control, embryonic liver obtained at E16.5, YAP was found to be localized in nuclei of the cells that made up the ductal plate (which give rise to intrahepatic bile ducts after birth), while phosphorylated YAP (S127) was localized to the cytoplasm of ductal plate cells and observed more sparsely in the cytoplasm of all cells throughout the liver (Fig. 3a and Supplementary Fig. 4a). In contrast, the levels of nuclear active YAP/TAZ were strongly increased in L1L2_Alb livers, but the phosphorylation level of YAP was diminished (Fig. 3a,b and Supplementary Fig. 2e). Consistent with this finding, L1L2_Alb livers displayed up-regulation of YAP signature genes including *Ankrd1, Ctgf* and *Cry61* (Fig. 3b–d, Supplementary Fig. 2f and Supplementary Table 2).

Based on the previous finding that liver-specific *Yap*-knockout mice failed to develop bile ducts^35^, we next tested whether YAP, TAZ or both were essential for the ductal plate cell expansions observed in L1L2_Alb livers. We found that *Yap/Taz* double-knockout mice showed more significant reductions of bile ducts (Supplementary Fig. 4b), suggesting that both YAP/TAZ are required for ductal plate cell proliferation and bile duct formation. Interestingly, mice that harboured either *Yap* or *Taz* (*Lats1*^−/−^*; Lats2*^*fl/fl*^*; Yap*^*fl/fl*^*; Taz*^*+/+*^*; Alb-Cre* or *Lats1*^−/−^*; Lats2*^*fl/fl*^*; Yap*^*+/+*^*; Taz*^*fl/fl*^*; Alb-Cre*) showed partially rescued phenotypes, but still exhibited abnormal liver development (Fig. 3e) and died within 1 month of birth. Only complete *Yap/Taz* deletion yielded a lack of ducts and complete rescue of the L1L2_Alb phenotype (Fig. 3f), indicating that one copy of *Yap* or *Taz* confers sufficient activity to promote ductal plate cell expansion in *Lats1/2*-deficient livers. These results collectively suggest that YAP and TAZ are both required for ductal plate formation and its proliferation, and that their hyper-activation by ablation of *Lats1/2* in embryonic livers enhances ductal plate cell proliferation.

The overexpression of YAP in our *in vitro* system yielded a phenocopy of *Lats1/2* deletion. In addition, the degrees to which BEC and hepatocyte differentiation were enhanced and suppressed, respectively, were modulated by YAP activity (Fig. 3g,h), potentially accounting for the expansion of biliary/progenitor cells in liver-specific *Mst1/2* double knockout and *Nf2* knockout livers (Fig. 3i). These results suggested that the increased YAP activity triggered by deletion of Hippo pathway components preferentially promotes the differentiation of hepatoblasts to BECs and increases their proliferation.

### Hyper-active YAP/TAZ up-regulates TGFβ signalling

**Figure 4:**
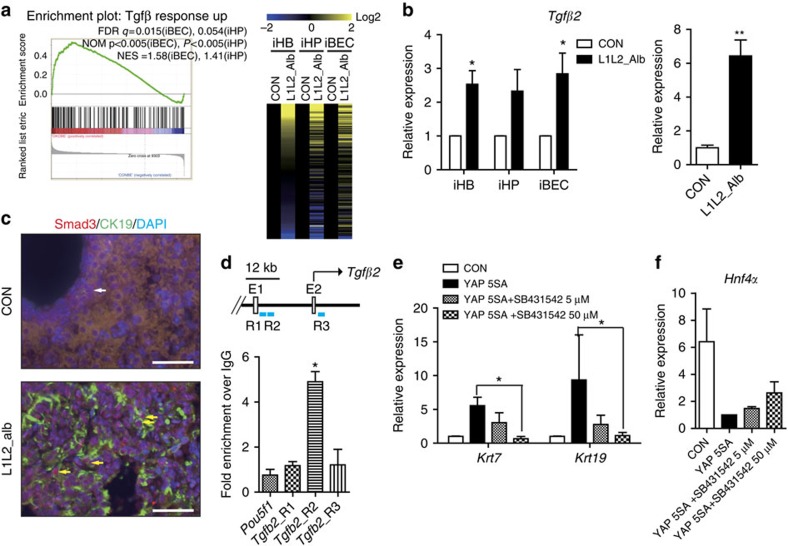
*Lats1/2*-deletion-induced YAP/TAZ activation activates TGFβ signalling. (**a**) A signature plot of *Tgfβ* response up-regulated gene set between *Lats1/2*-deficient and control iBECs, and heat map of mRNA expression levels of *Tgfβ* response up-regulated genes in control and *Lats1/2*-deleted HBs, iBECs and iHPs. (**b**) Relative *Tgfβ2* mRNA expression in HBs, iHPs and iBECs (left) and P1 livers of control and L1L2_Alb mice (right). (**c**) Representative immunofluorescence staining results obtained with anti-SMAD2/3 and CK19 in P1 livers. Yellow arrows indicate nuclear SMAD2/3 in CK19-positive cells and white arrow indicates cytoplasmic SMAD2/3 in CK19-positive cells. (**d**) Chromatin immunoprecipitation (ChIP) analysis of the *Tgfβ2* locus in flag-YAP 5SA over-expressing iHPs, as performed with anti-FLAG or –YAP. (**e**) Relative mRNA expression levels of *cytokeratin 7* and *19* in YAP 5SA over-expressing iBECs treated with or without the TGFβ receptor inhibitor, SB431542. (**f**) Relative mRNA expression levels of *Hnf4α* in YAP 5SA over-expressing iBECs treated with and without SB431542. Scale bars, 50 μm; the data are presented as means±s.e.m.; *n*=3, *P<0.05 and **P<0.01 (Student's *t*-test).

Our comparative analysis suggests that, during liver development, Hippo-YAP/TAZ signalling controls liver cell specification by regulating several pathways other than Notch signalling. In addition to JAG1, which activates Notch signalling to induce BEC differentiation, TGFβ is secreted from mesenchymal cells around the portal vein, where it induces differentiation into BECs (refs 37, 38), then TGFβ signalling is diminished during bile duct morphogenesis^30^. Consistently, TGFβ-up-regulated livers show expansion of immature BECs (ref. 39). Strikingly, we found that certain sets of TGFβ-responsive genes were significantly enriched in *Lats1/2*-deleted iHBs, iBECs and iHPs (Fig. 4a and Supplementary Data 1). Moreover, the mRNA expression of *Tgfβ2* was significantly elevated among *in vitro* differentiated cells and L1L2_Alb livers (Fig. 4b). We also observed that SMAD2/3 was localized to the nuclei of expanded BECs, indicating that TGFβ signalling was activated in L1L2_Alb livers (Fig. 4c). To define whether YAP directly regulates the transcription of *Tgfβ2*, we searched the *Tgfβ2* locus for TEAD-binding sequences which are recognized by YAP-TEAD complexes. Chromatin immunoprecipitation experiments revealed that YAP bound to the *Tgfβ2* locus (+9.2 kb; Fig. 4d). Importantly, we observed that treatment with 50 μM of the TGFβ receptor inhibitor, SB431542, significantly suppressed the BEC differentiation of YAP 5SA over-expressing hepatoblasts (Fig. 4e) and slightly restored the expression levels of *Hnf4α* and *Cyp7a1* in hepatocytes over-expressing the YAP 5SA mutant (Fig. 4f). These results suggest that activation of TGFβ signalling might be a main mechanism through which BEC commitment, proliferation and defective bile duct morphogenesis are enhanced in *Lats1/2*-deficient hepatoblasts and embryonic liver.

### *Lats1/2* deletion triggers transition of hepatocytes to BECs

**Figure 5:**
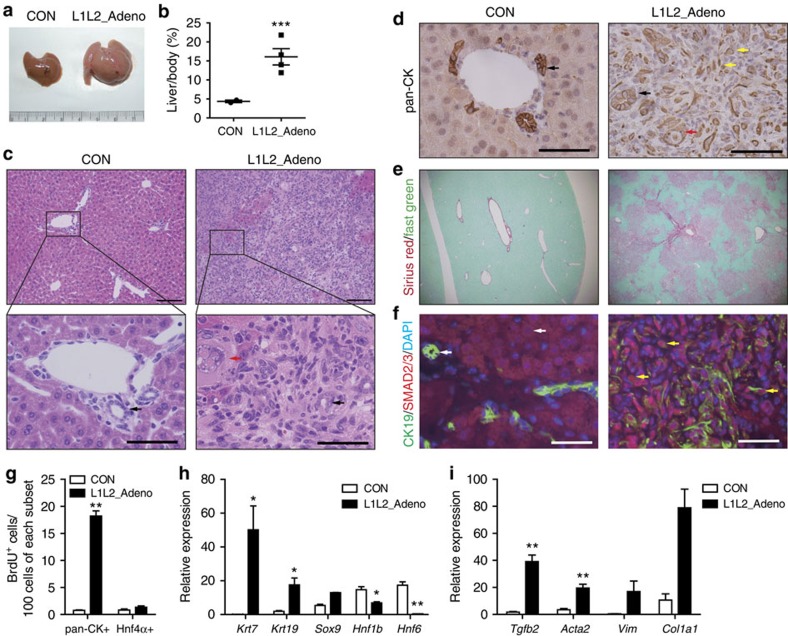
Loss of *Lats1/2* in adult livers causes rapid expansion of immature BECs and activation of TGFβ signalling. (**a**,**b**) Photograph (**a**) shows livers of adeno-Cre (2X10^8^ PFU)-injected control and *Lats1^*−/−*^; Lats2^fl/fl^* mice (L1L2_Adeno) and dot graph (**b**) of liver to body weight ratio of adeno-Cre virus injected livers. (**c**–**f**) Representative images of haematoxylin and eosin (H&E) (**c**), anti-pan-CK (**d**), Sirius red and Fast green (**e**) and, anti-SMAD2/3 and anti-CK19 (**f**) staining of control and L1L2_Adeno livers. Black arrows indicate tubular bile ducts and yellow arrows indicate immature BECs. Red arrows indicate LCCs in hepatocytes. Scale bars in (**c**) up indicate 100 μm and scale bars in (**c**) bottom indicate 50 μm. Yellow arrows in **d** indicate pan-CK-positive signal. White arrows in **f** indicate cytoplasmic SMAD2/3 signal in CK19-positive cells; Yellow arrows in **f** indicate nuclear SMAD2/3 signals in CK19-positive cells. Scale bars in **d** and **f** indicate 50 μm. (**g**) Bar graph showing quantification of BrdU-positive cells out of 100 pan-CK- and HNF4α-positive cells. (**h**) Relative mRNA expression levels of BEC-related genes in control and L1L2_Adeno livers. *n*=4. (**i**) Relative mRNA expression levels of *Tgfβ2* and fibrotic markers in control and L1L2_Adeno livers. The data are presented as means±s.e.m.; *n*=4, *P<0.05, **P<0.01 and ***P<0.005 (Student's *t*-test).

Next, we questioned whether deletion of *Lats1/2* after liver development could also enhance TGFβ2 secretion in adult livers, leading to the expansion of immature BECs, the de-differentiation of hepatocytes into biliary/progenitor cells and/or hepatic tumour development, as reported in other mouse models subjected to Hippo component knockout and YAP over-expression^9^,^19^,^20^,^21^,^22^,^23^,^24^. We thus performed acute deletion of *Lats1/2* in adult livers with adeno-Cre (L1L2_Adeno) or AAV-TBG-Cre (L1L2_AAV) viruses. L1L2_Adeno mice showed abdominal distension, hepatomegaly and eventual lethality caused by liver failure (Fig. 5a,b and Supplementary Fig. 5a,b). L1L2_Adeno livers displayed expansion of non-hepatocyte cells, including immature BECs, fibroblasts and immune cells, within 2–3 weeks after adeno-Cre virus infection (Fig. 5c–e and Supplementary Fig. 5c). The pan-CK-positive cells in the L1L2_Adeno livers were highly proliferative, but the hepatocytes were not (Fig. 5g and Supplementary Fig. 5d–f). This phenotype was remarkably similar to that of the L1L2_Alb livers, but distinct from that of other Hippo pathway-knockout livers.

We next wanted to determine the origin of the over-abundant immature BECs, whether they arose from resident bile duct cells or de-differentiated from hepatocytes. To do this, we used the AAV-TBG-Cre virus (5 × 10^11^−1 × 10^12^ gene copy number per mouse). This is because AAV-TBG-Cre specifically infects hepatocytes^27^. These L1L2_AAV mice also became ill within 2 weeks because of hepatic failure (Supplementary Fig. 6a). As with the L1L2_Adeno livers, the L1L2_AAV livers were filled with CK19-positive BECs and had few hepatocytes (Supplementary Fig. 6b–d). By performing recombination PCR on the hepatocyte and non-parenchymal cell fractions from L1L2_AAV livers, we found that roughly 60% of the immature BECs arose from *Lats1/2*-deficient hepatocytes (Supplementary Fig. 6e). Thus, most of the immature BECs arose from *Lats1/2*-deleted hepatocytes via de-differentiation, and non-cell autonomous factors seem to have driven their expansion.

To explore the involvement of Notch signalling in this de-differentiation, we generated *Lats1*^−/−^*; Lats2*^*fl/fl*^*; Notch2*^*fl/fl*^ (L1L2N2_AAV) mice and injected them with AAV-TBG-Cre viruses. These L1L2N2_AAV mice showed a similar phenotype to L1L2_AAV mice, including the BEC expansion (Supplementary Fig. 6f–h), suggesting that at least NOTCH2 does not involve in this de-differentiation. Consistent with the finding that YAP overexpression is sufficient to de-differentiate hepatocytes into biliary/progenitor cells in mice^27^, our results indicate that the LATS kinases restrict immature BEC proliferation of and prevent the transition of mature hepatocytes into immature BECs in adult livers by direct inhibition of YAP/TAZ.

Notably, L1L2_Alb and L1L2_Adeno livers showed drastically enhanced BEC commitment but did not undergo bile duct morphogenesis (Figs 2a–c and 5c,d). Consistent with these findings, L1L2_Adeno livers showed high expression levels of *cytokeratin 7* and *19*, but diminished expression of *Hnf6* and *Hnf1β* (two key molecules in bile duct morphogenesis). This is similar to the results we observed in L1L2_Alb livers (Figs 2g and 5h). We also confirmed that SMAD2/3 was localized in the nuclei of L1L2_Adeno liver cells (Fig. 5f and Supplementary Fig. 5g), and that the mRNA expression levels of *Tgfβ2* and its fibrosis-related target genes were significantly increased in L1L2_Adeno livers (Fig. 5i). The up-regulation of TGFβ signalling in L1L2_Adeno livers by YAP/TAZ may enhance recruitment and proliferation of fibrocytes and promote collagen-fiber production, thereby inducing fibrosis. Thus, we suggest the activation of YAP/TAZ caused by loss of *Lats1/2* up-regulates TGFβ signalling to induce immature BEC expansion and fibrosis in adult livers.

### YAP/TAZ hyper-activation in hepatocytes induces senescence

Since TGFβ signalling inhibition did not completely rescue the inhibition of hepatic differentiation caused by YAP 5SA overexpression, we thought YAP/TAZ might regulate hepatic differentiation via another signalling pathway. Indeed, we found that although the hepatocytes of *Lats1/2*-deficient embryonic and adult livers had nuclear YAP, they did not show hepatocyte hyper-proliferation (Figs 2h,i and 5g and Supplementary Fig. 5e). For this reason, we next asked whether the reduced hepatocyte differentiation was caused by a defect in hepatocyte proliferation or a problem with differentiation itself.

**Figure 6:**
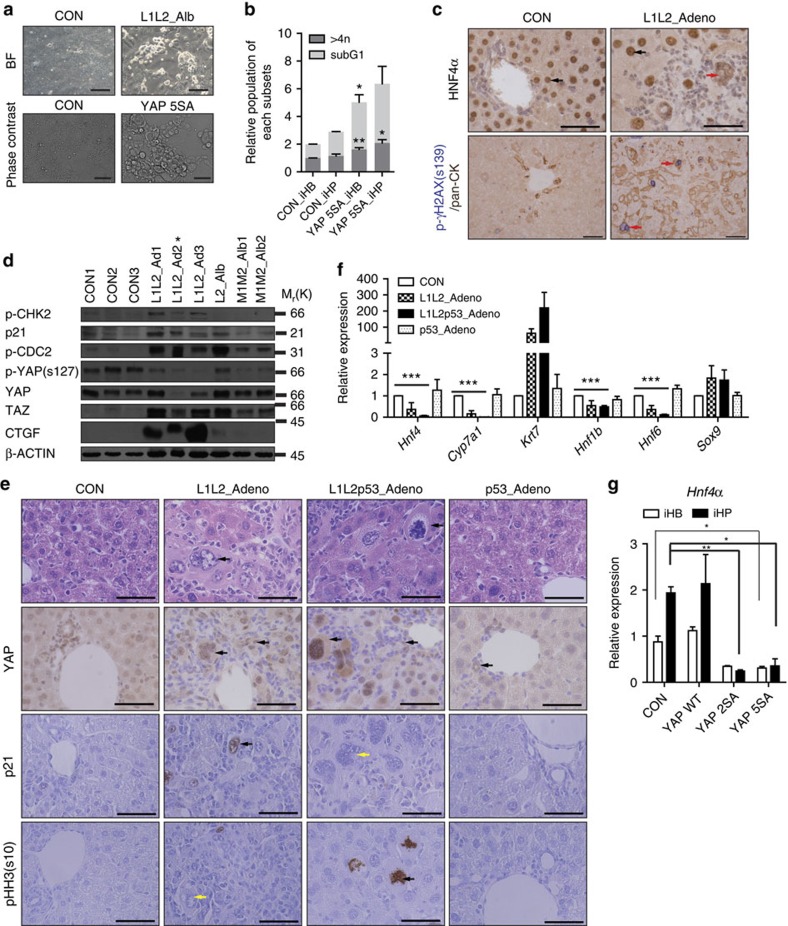
Hyper-activation of YAP induced oncogenic stress-induced senescence or apoptosis, which does not affect hepatocyte differentiation. (**a**) Representative bright-field (upper) and phase contrast (lower) images of *in vitro* differentiated hepatocytes from control cells, L1L2_Alb hepatoblasts (upper, scale bars, 50 μm) and YAP 5SA over-expressing hepatoblasts (lower, scale bars, 100 μm). (**b**) fluorescence-activated cell sorting (FACS) analysis of *in vitro* cultured hepatoblasts and hepatocytes. *n*=3. (**c**) Representative immunohistochemical results obtained with anti-HNF4α (upper) and anti-phospho-γH2ax (S139) (lower) in control and L1L2_Adeno livers. Black arrows indicate normal hepatocytes; red arrows in (**c**) up indicate LCCs of hepatocytes and red arrows in (**c**) bottom indicate positive signal to p- γH2AX. Scale bars, 50 μm. (**d**) Western blot analysis of liver extracts from control and *Lats1^*−/−*^; Lats2^fl/fl^* mice injected with adeno-Cre (L1L2_Ad; lanes 1–6), *Lats2^fl/fl^; Alb-Cre* (L2_Alb; lane 7, HCC) and *Mst1^fl/fl^;Mst2^*−/−*^;Alb-Cre* (Mst1/2_Alb; lanes 8 and 9, HCC). Asterisk indicates the lysate from livers with highly expanded fibrocytes. (**e**) Representative images of haematoxylin and eosin (H&E) staining and immunohistochemistry with antibodies against YAP, p21 and pHH3 (S10) in control, L1L2_Adeno, L1L2_p53_Adeno and p53_Adeno livers. In the H&E staining images, black arrows indicate LCCs of hepatocytes. In the immunohistochemical staining images, black arrows indicate positive signal to indicated antibodies and yellow arrows indicate a lack of staining. Scale bars, 50 μm. (**f**) Relative mRNA expression of hepatic and BEC markers in livers from mice of the indicated genotypes. (**g**) Relative *Hnf4α* mRNA expression levels of control, YAP WT, -2SA and -5SA over-expressing iHPs. The data are presented as means±s.e.m.; *n*=3, *P<0.05, **P<0.01 and ***P<0.005 (Student's *t*-test).

First, in the RNA-sequencing data from *Lats1/2*-deficient iHPs, we observed p53 pathway activation and E2F target gene down-regulation (Supplementary Fig. 7a and Fig. 1c). Together, these suggested an increase in DNA damage and a decrease in proliferation. In addition, we noticed that *Lats1/2*-deficient or YAP 5SA over-expressing iHPs seemed to have a distinctly bulgy morphology (Fig. 6a). Fluorescence-activated cell sorting analysis further confirmed an increased frequency of polyploid and apoptotic cells among YAP 5SA over-expressed iHBs and iHPs compared with controls (Fig. 6b and Supplementary Fig. 7b–d). Interestingly, some of the mature hepatocytes in *Lats1/2*-deleted adult livers showed bizarre cell shapes and at least two-fold enlargements of the cytoplasm and nucleus (the so-called ‘large cell change' (LCC), a kind of hepatocyte dysplasia^40^), compared with normal hepatocytes (Fig. 6c up, red arrow). In another fluorescence-activated cell sorting analysis of hepatocyte fractions, we found L1L2_Adeno liver hepatocytes had higher DNA content than control hepatocytes (Supplementary Fig. 7e). In contrast to the highly proliferative BECs in the same livers, these enlarged hepatocytes (∼80% showing LCC) were positive for p21, phospho-γ-H2AX and TUNEL (Fig. 6c and Supplementary Fig. 7f,g). Unlike *Mst1/2* double-knockout HCC cells, *Lats1/2*-deleted livers clearly showed increased levels of p21 expression and CHK2 phosphorylation, suggesting the presence of DNA damage and p53 activation (Fig. 6d). A similar phenotype was reported in livers expressing the oncogene, H-rasG12V (ref. 41). Moreover, the hyper-activation of YAP/TAZ induced by *Lats1/2* deletion or YAP 5SA overexpression was found to induce polyploidy in mouse embryonic fibroblasts (MEFs), triggering DNA damage and activating p53-dependent cell death (Supplementary Fig. 7h,i). This notion was further supported by our observation that deletion of p53 rescued the viability and proliferation capacity of *Lats1/2*-deficient liver cells and MEFs (Fig. 6e and Supplementary Fig. 8a–c).

However, we still observed the transition of hepatocytes into immature BECs and the expansion of immature BECs in *Lats1*, *Lats2* and *p53* triple-knockout livers (L1L2p53_Adeno Fig. 6f). Moreover, the YAP/TAZ hyper-activation in *p53*-deficient hepatoblasts still suppressed the differentiation into hepatocytes (Fig. 6g). We therefore concluded that the apoptosis or growth arrest induced by YAP/TAZ activation did not affect hepatocyte specification.

### YAP/TAZ suppress hepatic differentiation by repressing *Hnf4α*

**Figure 7:**
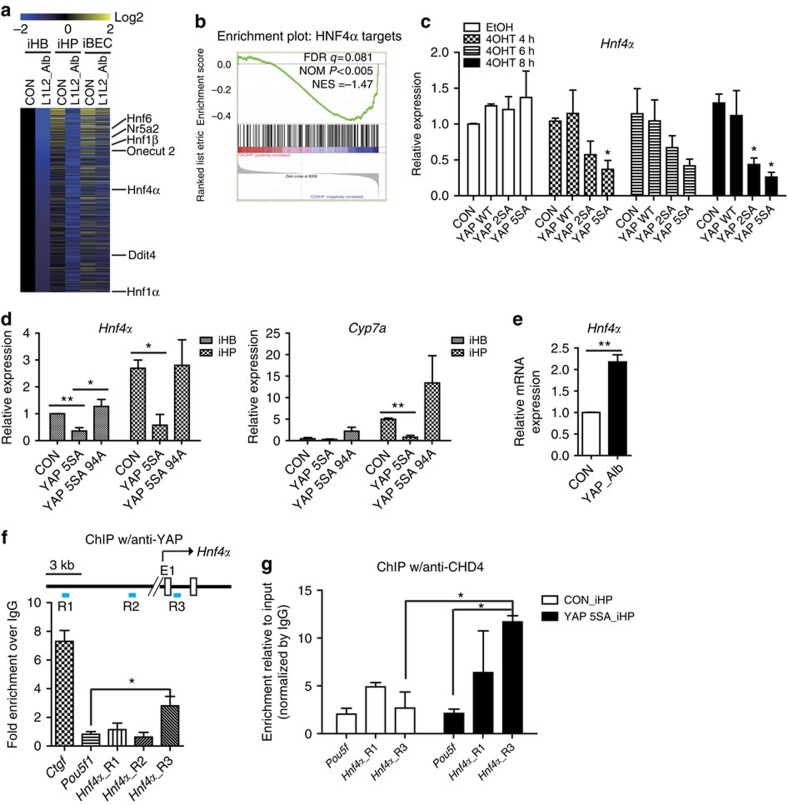
YAP represses the hepatic transcription factor *Hnf4α*, resulting in defective hepatocyte differentiation. (**a**) Heat map for genes found to be commonly down-regulated in *Lats1/2*-deficient hepatoblasts, differentiated hepatocytes and BECs. (**b**) *Hnf4α* target gene signature plot between control and *Lats1/2*-deficient iHPs. (**c**) Relative mRNA expression levels of *Hnf4α* in Aml12 cell lines stably expressing oestrogen receptor fused Yap mutants; control, YAP WT, -2SA and -5SA-ER^T2^ following their induction by tamoxifen for the indicated times. *n*=2. (**d**) Relative mRNA expression levels of *Hnf4α* and *Cyp7a1* in YAP 5SA and YAP 5SA 94A (Tead-binding defective) over-expressing iHBs and iHPs. (**e**) Relative mRNA expression levels of *Hnf4α* in control and Yap-knockout iHPs. (**f**,**g**) chromatin immunoprecipitation (ChIP) analysis of the *Hnf4α* locus in flag- YAP 5SA over-expressing iHPs, performed using anti-flag or -YAP (**f**) and anti-CHD4 (**g**). The data are presented as means±s.e.m.; *n*=3, *P<0.05 (Student's *t*-test).

Because the defective hepatic differentiation in YAP 5SA over-expressing cells was not entirely rescued by inhibition of TGFβ signalling or the p53 pathway (Figs 4f and 6g), we speculated that YAP/TAZ might modulate hepatic differentiation via another signalling pathway. It is well-documented that there is a hepatic transcription factor network in which *Hnf1α*, *Hnf1β*, *Hnf4α*, *Foxa2*, *Hnf6* and *Nr5a2* regulate the expression levels of each other and various hepatic genes^42^. Indeed, during liver development, HNF4α plays a most critical role in regulating other hepatic transcription factor network components^42^. Recent studies have suggested that YAP cooperates with HNF4α and FOXA2 in the enhancer regulatory mechanisms of the liver and pancreas^43^,^44^. Thus, we hypothesized that hyper-activated YAP could affect hepatic differentiation by altering the activities of HNF4α and FOXA2. However, most of the network genes and their targets (except *Foxa2*) were down-regulated in *Lats1/2*-deleted iHPs, iBECs and even undifferentiated hepatoblasts (Fig. 7a,b and Supplementary Data 2). Moreover, *Hnf4α* expression decreased rapidly after the induction of YAP 5SA in hepatoblasts, iHPs and the mature mouse hepatocyte cell line, Aml12 (Fig. 7c,d). Overexpression of the Tead-binding-defective YAP 5SA 94A mutant did not suppress *Hnf4α* expression in hepatoblasts and induced normal hepatic differentiation indicating that TEAD proteins are required for this process (Fig. 7d). Indeed, *Yap* deficiency enhanced the expression of *Hnf4α* (Fig. 7e). Thus, the observed decreases in the expression levels of hepatic transcription factors in *Lats1/2*-deficient and YAP 5SA over-expressing hepatoblasts appear to reflect direct regulation by YAP rather than being downstream of the differentiation defects. While YAP/TAZ are known to be transcriptional co-activators capable of up-regulating target genes, they also play repressive roles in cooperation with repressor complexes^45^,^46^,^47^. We found several Tead-binding motifs in the *Hnf4α* locus, and subsequently tested whether YAP could directly repress *Hnf4α* expression. Our chromatin immunoprecipitation results showed that the *Hnf4α* locus (TSS+0.5 kb) was enriched by YAP (Fig. 7f). Furthermore, CHD4, a repressor complex that interacts with YAP (ref. 47) also enriched the same region of the *Hnf4α* locus (Fig. 7g). Based on the results presented above, we conclude that YAP/TAZ control hepatic differentiation by directly repressing the hepatic transcription factor network.

## Discussion

YAP/TAZ have been proposed as differentiation regulators that determine the specific lineages obtained from various progenitors. Here, we focused on the roles of YAP/TAZ in liver development and maintenance, using *Lats1* and *Lats2* double-knockout mice. The Hippo-YAP/TAZ axis differentially affects cell fates and properties in the liver in a cell-type-specific manner. LATS1/2-mediated inhibition of YAP/TAZ is required to restrict the commitment and proliferation of immature BECs during liver development and in the adult liver. It also acts in the adult liver to promote hepatic differentiation and maintain hepatocytes in the quiescent state.

Many studies have suggested that the Hippo pathway regulates Notch signalling in the differentiation of progenitors and in the progression of cancer in the liver, pancreas and intestine^48^,^49^,^50^. Our study, however, indicates Notch signalling activation is not responsible for the BEC expansion observed with endogenous YAP/TAZ activation induced by Hippo pathway disruption. We show for the first time that the YAP/TAZ activation triggered by loss of *Lats1/2* during liver development leads to immature BEC expansion. Although *Lats1/2*-deficient mouse livers phenocopy some aspects of N1ICD transgenic livers (for example, BEC expansion), there are important differences. While *Lats1/2* deficiency causes a disorganized expansion of immature BECs, N1ICD overexpression leads to the excessive formation of differentiated bile ducts. Also, while the bile duct expansion induced by N1ICD overexpression depends on SOX9 (ref. 46), *Lats1/2* deficiency does not increase *Sox9* expression. These differences suggest YAP/TAZ and Notch activate BEC expansion via distinct mechanisms.

This is consistent with our finding that *RBPJ* deficiency only modestly restricts BEC expansion in *Lats1/2*-deficient livers (Fig. 2e). Additionally, YAP deficiency significantly reduces abnormal bile duct expansion even in the context of activated Notch signalling of N1ICD over-expressing embryonic livers (Supplementary Fig. 3b–d). It will be interesting to see if *Yap/Taz* double knockout causes further reduction.

Based on our findings and previous reports, NOTCH-SOX9 and Hippo-YAP signalling are both required for embryonic bile duct development, but seem to act in parallel. Activation of YAP/TAZ enhances BEC characteristics and induces the proliferation of immature BECs; activation of NOTCH enhances BEC commitment and induces the morphogenesis of bile ducts. Future studies will determine how the Notch and Hippo-YAP signalling pathways interact with one another during bile duct development. It will also be interesting to determine whether YAP/TAZ are involved in the postnatal bile duct formation of *Notch*-deficient livers and vice versa.

Studies on the crosstalk between the TGFβ and Hippo signalling pathways have focused mainly on the interaction between SMAD and YAP/TAZ in target gene regulation. Here, in the context of *Lats1/2*-deficient livers, we demonstrated transcriptional up-regulation of *Hnf4α* and activation of TGFβ signalling induced by YAP/TAZ. These changes promote the differentiation of hepatoblasts into immature BECs. Most work on TGFβ signalling in the adult liver has focused on its role in fibrosis and HCCs. Our finding that the hyper-activation of YAP/TAZ triggered by loss of *Lats1/2* forces the transition of mature hepatocytes into immature BECs suggests TGFβ signalling may also regulate this transition in *Lats1/2*-deficient embryo livers. Remarkably, *Lats1/2*-deficient livers show severe fibrosis accompanied with the ductal reaction associated with various hepatic diseases. We assume that, together with an up-regulation of TGFβ signalling, secreted factors like *Ctgf* and *Timp2*, which are induced by YAP/TAZ, accelerate fibrosis in *Lats1/2*-deleted livers. This likely occurs via recruitment and activation of fibrocytes/immune cells/hepatic stellate cells, which accumulate extracellular matrix components like collagens^51^,^52^.

Interestingly, we observed that some of the hepatocytes remaining in *Lats1/2*-deficient adult livers showed LCCs characterized by cellular enlargement, nuclear pleomorphism and multinucleation^40^. These LCCs are frequently observed in in chronic human liver diseases, such as cirrhosis, and in the livers of some transgenic mice expressing oncogenic proteins (for example, CyclinD1-, β-Catenin/H-rasG12V- and c-Myc/Tgfα) before their development of HCC (ref. 40). Particularly, H-rasG12V alone led to LCCs in hepatocytes, but the resulting dysplastic hepatocytes could not be expanded clonally due to oncogene induced senescence^41^. Similarly, loss of *Lats1/Lats2* produced dysplastic hepatocytes that were senescent rather than proliferative. Previous studies have shown that LATS1/2 regulate p53 stability in response to DNA damage and that YAP/TAZ are cytoprotective against DNA damage^53^,^54^,^55^. We speculate, though, that either *Lats1/2* deletion or YAP 5SA overexpression induces an oncogenic activation and stress. This oncogenic stress-induced DNA damage would increase p53 activity in primary hepatocytes. The fact that deletion of p53 rescues the senescence and apoptosis phenotypes of *Lats1/2*-deficient MEFs and livers supports our hypothesis that *Lats1/2*-deletion-induced hyper-activation of YAP triggers p53-dependent senescence in senescence-prone cells.

These two distinct hepatocyte phenotypes in L1L2_Adeno livers may arise from differences in the initial status of the hepatocytes. During postnatal liver growth, some proliferating hepatocytes fail to complete cytokinesis, producing multinucleated cells^56^. In addition, hepatocytes in different hepatic zones encounter different levels of oxygen and toxic materials and as a result show different gene express profiles^57^. On the other hand, some hepatocytes arise from ductal plate cells that do not undergo final duct morphogenesis and these cells often de-differentiate^5^,^30^. Thus, while some hepatocytes are prone to polyploidization, others are prone to de-differentiation. Therefore, even if *Lats1/2*-deficient hepatocytes have an active p53 pathway and active TGFβ signalling, different subsets of hepatocytes may behave differently. Consistent with this idea, we were unable to find LCCs among hepatocytes in L1L2_Alb livers because most hepatoblasts and immature hepatocytes were becoming immature BECs.

In MEFs, which do not need to change their cellular characteristics, both YAP 5SA overexpression and *Lats1/2* deletion generally induce polyploidization, senescence and apoptosis. Of the tested mutants of YAP, only overexpression of YAP 5SA also caused growth arrest and enlargement of hepatocytes and MEFs. This could explain why only YAP 5SA over-expressing hepatocytes by hydrodynamic injection in adult mice disappeared within a few days, as mentioned in a previous report^58^.

In L1L2_Adeno livers, most of the immature BECs were CK19-positive and EpCAM-negative, with only a few positive for both markers. This result differs from the observation in a previous report^27^ that hepatocyte overexpression of S127A YAP caused de-differentiation into cells positive for both CK19 and EpCAM. Additionally, cytokeratin expression in iBECs rises in accordance with increasing YAP activation via phosphorylation. Based on our results, YAP/TAZ appear to act in the liver as a switch between BEC and hepatocyte specification; the activation of YAP/TAZ induces a shift in both liver progenitor cells and mature cells towards a BEC phenotype, and the extent to which this change to the BEC phenotype occurs depends on the level of YAP/TAZ activation. This is consistent with our observation that Mst1/2_Alb, Sav1_Alb, Nf2_Alb and Lats1/2_Alb livers show different BEC-to-hepatocyte ratios, reflecting differences in the phosphorylation status (and thus activity) of YAP among the different lines. The highest YAP activity—found in *Lats1/2*-deficient mice—is associated with the highest levels of immature BEC expansion, hepatocyte to immature BEC transitions, and hepatic LCCs. The lower YAP activity observed in *Sav1*-deficient cells causes increased biliary/progenitor cell proliferation without altering hepatocyte growth, perhaps indicating that YAP differentially regulates the differentiation and proliferation of each cellular subset in this model. Thus, we conclude that although each of these lines lack components of the Hippo pathway, their distinct pathway disruptions lead to unique changes in hepatocyte-like, progenitor-like or BEC-like cellular populations. This may explain the wide range of phenotypes observed in all the different Hippo pathway-knockout livers^19^,^20^,^22^,^23^,^24^,^25^,^26^.

Recently, the siYap-LNP-mediated inhibition of YAP in advanced HCC was shown to reduce tumours by regulating proliferation and hepatic differentiation^59^. Additionally, YAP activation in some populations of hepatocytes is reportedly insufficient to trigger clonal expansion in the absence of a second signal induced by liver damage^60^. However, it remains unknown how hepatic markers are reduced by YAP/TAZ activation in response to liver injury. Our findings provide evidence that YAP/TAZ directly up-regulate TGFβ signalling and suppress hepatic differentiation by reducing the mRNA expression levels of hepatic transcription factor network. In terms of repression, YAP/TAZ were previously shown to affect chromatin remodelling complexes^45^,^47^, and a complex containing SMAD, YAP/TAZ was recently proposed to regulate the transcription of their target genes^61^. Thus, the YAP/TAZ-mediated repression of the hepatic transcription factor network may involve the cooperation of SMAD. Our study suggests for the first time that these two additional mechanisms which include up-regulation of *Tgfβ* expression and down-regulation of hepatic transcription factor network expression yield distinct features (for example, fewer mature bile ducts and more fibrocytes) compared with those observed following the up-regulation of Notch signalling.

Based on our work, we propose that the fine-tuning of endogenous YAP/TAZ activity is a key aspect of cell fate determination in the liver. To restrict bile duct expansion and promote hepatocyte differentiation, LATS1/2 and other Hippo pathway components must undergo specific spatiotemporal regulation. During bile duct development and morphogenesis, it is known that mesenchymal-epithelial cell interaction are required for normal duct formation^62^. Furthermore, YAP/TAZ activation induces the secretion of ECM related factors such as CTGF, TGFβ, LAMININ and COLLAGEN IV that are important for duct formation^6^ and in stiffening the surrounding ductal plate. If YAP/TAZ are activated by mechanical forces, it would make sense that they would consequently promote a stiffening of the ECM. Future experiments will determine the signals that control the Hippo pathway in the context of location, cell type and ECM stiffness during and after liver development.

## Methods

### Mice

*Lats1^−/−^, Lats2^fl/fl^, Yap^fl/fl^, Taz^fl/fl^, Mst1^fl/fl^, Mst2^−/−^, Nf2^fl/fl^, Notch2^fl/fl^* and *p53^−/−^* mice were previously described^63^,^64^,^65^,^66^,^67^,^68^,^69^,^70^. Albumin-Cre (*B6.Cg-Tg(Alb-cre*)21Mgn/J), conditional NICD over-expressing (*Gt(ROSA)26Sor^tm1(Notch1)Dam^/J*) mice, and *Notch2^fl/fl^* mice were purchased from The Jackson Laboratory and *RbpJ^fl/fl^* mice were obtained from RIKEN (RBRC01071). To delete *Lats2* allele, 2 × 10^8^∼2 × 10^9^ PFU of Adeno-Cre virus (Vector Bio 1710, 1710-HT) was diluted in PBS and intravenously injected (100 μl) into 4–6-week-old female mice (B6/129 mixed strain). AAV-TBG-Cre virus was purchased from (University of Pennsylvania Vector Core, AV-8-PV1091) or purified with AAV virus according to protocol (provided by the Salk Institute for Biological Studies). To purify AAV-TBG-Cre virus, we transfected 6 μg of each plasmids (pAAV2/8, pAdDeltaF6 and pENN.AAV.TBG.PI.Cre; purchased from University of Pennsylvania Vector Core) with PEI to 293T cells on 100 mm plate. Seventy-two hours after transfection, cells from 20 plates were harvested and centrifuged at 1,000*g* for 10 min. The pellet was resuspended with 20 ml lysis buffers containing 0.15 M NaCl, 50 mM Tris-Cl (pH8.5), 0.05% Tween then resulting lysates were frozen in liquid nitrogen and thawed at 37 °C three times. Then benzonase was added to a final concentration of 50 Unit ml^−1^ and incubated at 37 °C for 30 min. Lysate was then centrifuged at 3,700*g* for 20 min and supernatant was transferred to a new tube. Next, 6 ml of AAV lysate was gently laid on the top of a iodixanol gradient (15%–25%–40%–54%) in Beckman Ultra Tubes (Ca.34241) and ultracentrifuged at 48,000 r.p.m. in a Beckman 70Ti rotor for 2 h. The purified AAV virus containing-layer was extracted with 5 ml syringe and 19 Gauge needle. Finally, AAV virus concentration and buffer exchange (into PBS) were performed with Amicon Ultra centrifugal fiters (Millipore UFC910024) at 3,700*g* for 20 min. We collected 100 μl of virus and for quantification of gene copy number, the viral genome was extracted with NaOH followed by qRT-PCR. Animal care and all experiments were performed in accordance with guidelines approved by the Biomedical Research Center of Korea Advanced Institute of Science and Technology.

### Histology, immunohistochemistry and immunofluorescence analysis

Embryonic livers were extracted from pregnant female or newborn mice at E14.5∼P1. Adult mice were killed and perfused with 20 ml PBS before liver isolation. Livers were fixed in PBS including 10% of formalin at 4 °C for 4–24 h and embedded in paraffin. Sections (4 μm) were cut and stained with haematoxylin and eosin. For immunohistochemistry and immunofluorescence staining, antigen retrieval was performed in 1 mM EDTA, pH 8.0 by boiling inside a cocker within a microwave. After cooling, blocking solution including 3% of BSA and 0.3% of Triton x-100 in PBS was treated for 1 h. For immunohistochemistry, we added one more step to inhibit endogenous peroxidase with 1% of H_2_O_2_ for 10 min. The sections were reacted with antibodies against YAP (Cell Signaling, 1:100), phospho-YAP (Cell Signaling (S127), 1:100), phospho-Histone H3 (Cell Signaling (S10), 1:200), SMAD2/3 (Santa Cruz, 1:100), BrdU (BD Bioscience, 1:400), p21 (BD Bioscience, 1:200), pan-CK (Dako, 1:200), Keratin 19 (DSHB (Troma III), 1:200) and HNF4α (Santa Cruz, 1:200) at 4 °C overnight. For immunohistochemistry, sections were washed with PBS and developed using DAB Substrate Kit (Dako), and then counterstained with haematoxylin. For immunofluorescent staining, sections were washed with PBS and incubated with anti-goat AF488, anti-rabbit AF594, anti-mouse AF488, anti-rabbit AF488 (Invitrogen), and then counterstained with 4′,6-diamidino-2-phenylindole (Sigma). Images were obtained using an Imager M1 fluorescence microscope (Zeiss), an Axio Scan Z1 (Carl Zeiss) or a Leica DMLB microscope (Leica).

### Hepatoblast isolation and *in vitro* differentiation

For hepatoblast culture, control and L1L2_Alb livers were dissected and digested in 1 mg ml^−1^ collagenase/dispase (Roche) for 30 min at 37 °C with constant shaking. Total cells were passed through a 100-μm cell strainer (BD Bioscience) and centrifuged at 250*g* for 10 min. Red blood cells were lysed with 1X Ammonium-Chloride-Potassium buffer and cells were counted. The hepatoblasts (1 × 10^6^) were plated on collagen I- (BD Bioscience) or gelatin- (Sigma) coated 60-mm dishes and cultured in Dulbecco's modified Eagle's medium/Nutrient Mixture F12 (DMEM/F12) supplemented with 10% foetal bovine serum (FBS), 25 ng ml^−1^ human epidermal growth factor (hEGF) (Peprotech), 25 ng ml^−1^ human hepatocyte growth factor (hHGF), 5 μg ml^−1^ insulin and 40 ng ml^−1^ dexamethasone (Sigma). For hepatic differentiation, cells were treated with 12.5 ng ml^−1^ Oncostatin M (Sigma) for 6 days. For BEC differentiation, hepatoblasts were cultured on Matrigel (BD Bioscience) for 14 days. For Yap overexpression, we infected retroviruses-containing vector, Yap WT, YAP 2SA and YAP 5SA 2 days after plating. The retroviruses were produced in 293 T cells with pMSCV puro, pMSCV-Yap WT, pMSCV-Yap 2SA, pMSCV-Yap 5SA and packaging vectors (Gag-pol and VSV-G).

### Immunoblot analysis

Whole livers and cell lines were lysed with radioimmunoprecipitation buffer (50 mM Tris-Cl, pH 7.5, 150 mM NaCl, 0.1% SDS, 0.5% sodium deoxycholate and 1 mM EDTA) containing protease and phosphatase inhibitors (1 mM NaF, 1 mM Na_3_OV_4_, PMSF, 2 μg ml^−1^ leupeptin and pepstatin; all purchased from Sigma) for 30 min at 4 °C. The protein concentration in each lysate was quantified with a Pierce BCA protein assay kit (Thermo Scientific). Blots were incubated with anti-LATS1 (Bethyl Laboratories, 1:2,000), anti-LATS2 (Cell Signaling, 1:1,000), anti-phospho-YAP (Cell Signaling (S127), 1:1,000), anti-YAP (Cell Signaling, 1:1,000), anti-TAZ (Cell Signaling, 1:1,000), anti-CTGF (Santa Cruz, 1:500), anti-β-ACTIN (Sigma, 1:5,000), anti-phospho-CHK2 (Cell Signaling, 1:500), anti-phospho-CDC2 (Cell Signaling, 1:500) and p21 WAF1/CIP1 (BD Bioscience, 1:500). We included uncropped blots in Supplementary Fig. 9.

### Mouse embryonic fibroblast isolation and cell culture

For MEF isolation, we isolated E14.5 embryos and removed the internal organs and head. The rest of bodies were chopped and trypsinzed with 0.1% of Trypsin/EDTA solution at 37 °C for 15 min. This solution was resuspended with DMEM and centrifuged at 1,000 r.p.m. for 5 min. The pellets were resuspended in 10 ml DMEM supplemented with 10% FBS (Gibco) and 2 mM glutamine (Invitrogen) and the mixtures were left for 5 min to remove cell aggregates. Then, single cells from one embryo were plated into a 100-mm culture plate. For deletion of floxed Lats2, cells were introduced with Cre-expressing retrovirus at passage 2 and for over-expressing Yap mutants, retroviruses-containing vector, YAP WT, YAP 2SA and YAP 5SA were infected at passage 2 with 8 μg ml^−1^ polybrene (Hexadimethrine bromide, Sigma). All experiments were performed before passage 5.

Aml12 cells were purchased from ATCC and cells were cultured in DMEM/F12 supplemented with 10% FBS (Gibco), 2 mM glutamine, 5 μg ml^−1^ insulin, 5 μg ml^−1^ transfferrin (Sigma), 5 ng ml^−1^ selenium (Sigma), and 40 ng ml^−1^ dexamethasone (Sigma). For over-expressing Yap mutants, retroviruses-containing vector, YAP WT-ER^T2^, YAP 2SA-ER^T2^ and YAP 5SA ER^T2^ were infected with 8 μg ml^−1^ polybrene and 2 μg ml^−1^ puromycin was treated at 48 h after infection. 1 nM hydroxytamoxifen (Sigma) was treated to activate ER^T2^ system for indicated time.

### Hepatocyte isolation and Flow cytometry

Mice were euthanized and perfused with 30 ml of PBS then 0.5 mg ml^−1^ of a collagenase/dispase mixture via the portal vein. Livers were dissected and chopped into small pieces. 1 mg ml^−1^ of the collagenase/dispase mixtures was added and incubated with shaking for 30 min at 37 °C. After harvesting the supernatant, 1 mg ml^−1^ of the collagenase/dispase mixture was newly added and incubated for 30 min at 37 °C with constant shaking. The supernatant was passed through a cell strainer (100 μm) and centrifuged at 50*g* for 5 min at 4 °C. The pelleted cells included the hepatocyte fraction. Dead cells and cell debris were removed by 25% percoll (1,200*g*, 20 min, 4 °C). Density gradient centrifugation (25% percoll and 50% percoll, 1,800*g*, 30 min, 4 °C) was performed with the supernatant to produce the non-parenchymal fraction.

Hepatoblasts, *in vitro* differentiated hepatocytes and MEFs were harvested, fixed in 70% ethanol at −20 °C, and stained with diamidino-2-phenylindole. 20,000–50,000 cells were recorded per sample. First, we set the gates to remove doublets and calculated the frequency of SubG1 cells. To calculate polyploidy, we excluded the SubG1 cells and doublets, then set the gates as shown in Supplementary Fig.7b. We used the BD LSRFortessa with FlowJo v.7 for analysis.

### BrdU incorporation assay

For the *in vivo* BrdU incorporation assay, mice were intraperitoneally injected with 100 mg kg^−1^ BrdU (Sigma) 1 h before killing.

### Gene expression analysis by qRT-PCR

Total mRNA was isolated from livers or cultured cells using Ribo-ex (Gene All), complementary DNA was synthesized from 3.5 μg of total mRNAs with Moloney murine leukemia virus (M-MLV) reverse transcriptase (Enzynomics), oligo dT_18_ and random hexamer. qRT-PCR was performed using CFX Connect (BioRad) and 2X SYBR green premix (Enzynomics). The sequences of the utilized oligonucleotides are presented in Supplementary Table 3.

### Microarray and RNA-sequencing analyses

Total mRNA was isolated from livers and cell lines using an RNeasy Mini Kit (Qiagen). Microarray analysis was performed using a MouseRef-8 v2.0 chip (Illumina), and RNA-sequencing was performed with Truseq RNA library prep kit v2 (Illumina) and Hi-seq 2000 equipment. All gene expression values from Microarray and RNA-sequencing were changed to log_2_ value and analysed further. The Multi-experiment Viewer programme was used to draw Heat maps. The relative expression profiles used to generate each heat map are presented in Supplementary Tables 1 and 2 and Supplementary Data 1 and 2. Microarray data were analysed by *P*<0.05. For RNA-sequencing analysis, Gene set enrichment analysis was performed with v5.0 of the Molecular Signature Database (http://www.broadinstitute.org/gsea/msigdb/index.jsp) and gene sets which were <0.025 FDR *q*-value were stated. Raw data are available in GEO under accession code GSE71872, GSE71873 and GSE71874.

### Chromatin immunoprecipitation

Cells were cross-linked with 1% formaldehyde for 15 min at room temperature and neutralized with 125 mM glycine for 5 min. Cells were harvested, the cytosolic fraction was removed and the nuclear fraction was resuspended with a buffer containing 50 mM Tris-Cl, pH 7.5, 1% SDS, 0.5% deoxycholate and 1 mM MgCl_2_. The mixture was sonicated using a Bioruptor for 20 min (pulses of a 30-sec sonication and a 30-sec rest) and then centrifuged. The DNA concentration of each lysate was measured using spectrometry, and 50 μg of lysate was diluted in 50 mM Tris-Cl, pH 7.5, 0.5% Triton X-100 buffers and precleared with Protein A/G agarose beads (GenDEPOT). For immunoprecipitation, anti-CHD4 (Abcam), anti-FLAG (Sigma), anti-YAP (generated by T. Kim) and IgG (Santa Cruz) were incubated with the lysates overnight at 4 °C. After that, protein A/G beads were added and genomic DNAs were eluted and purified according to Cell Signaling manufacturer's protocol. Quantitative PCR were performed using CFX Connect (BioRad) and 2X SYBR green premix (Enzynomics). Primer information is presented in Supplementary Table 3.

### Statistical analysis

Graphing and statistical analysis (paired two-tailed Student's *t*-test) were performed using the GraphPad Prism 5 software.

### Data availability

The Microarray and RNA-seq data that support the findings of this study have been deposited in the NCBI Gene Expression Omnibus (GEO) with the accession code ‘GSE71872 (http://www.ncbi.nlm.nih.gov/geo/query/acc.cgi?acc=GSE71872)', ‘GSE71873 (http://www.ncbi.nlm.nih.gov/geo/query/acc.cgi?acc=GSE71873)' and ‘GSE71874 (http://www.ncbi.nlm.nih.gov/geo/query/acc.cgi?acc=GSE71874)'.

## Additional information

**How to cite this article**: Lee, D.-H. *et al*. Lats-Yap/Taz controls lineage specification by regulating Tgfβ signalling and Hnf4α expression during liver development. *Nat. Commun.* 7:11961 doi: 10.1038/ncomms11961 (2016).

## Supplementary Material

Supplementary Information Supplementary Figures 1-9 and Supplementary Tables 1-3

Supplementary Data 1 Gene lists of Heat map related to Figure 4a

Supplementary Data 2 Gene lists of Heat map related to Figure 7a
