# Supplementary material for: LATS-YAP/TAZ controls lineage specification by regulating TGFβ signaling and Hnf4α expression during liver development
Source: Nat Commun. 2016 Jun 30;7:11961. doi: 10.1038/ncomms11961 (PMC4931324; doi:10.1038/ncomms11961)
Supplement: Supplementary Information — Supplementary Figures 1-9 and Supplementary Tables 1-3 [file ncomms11961-s1.pdf]

Supplementary Figure 1

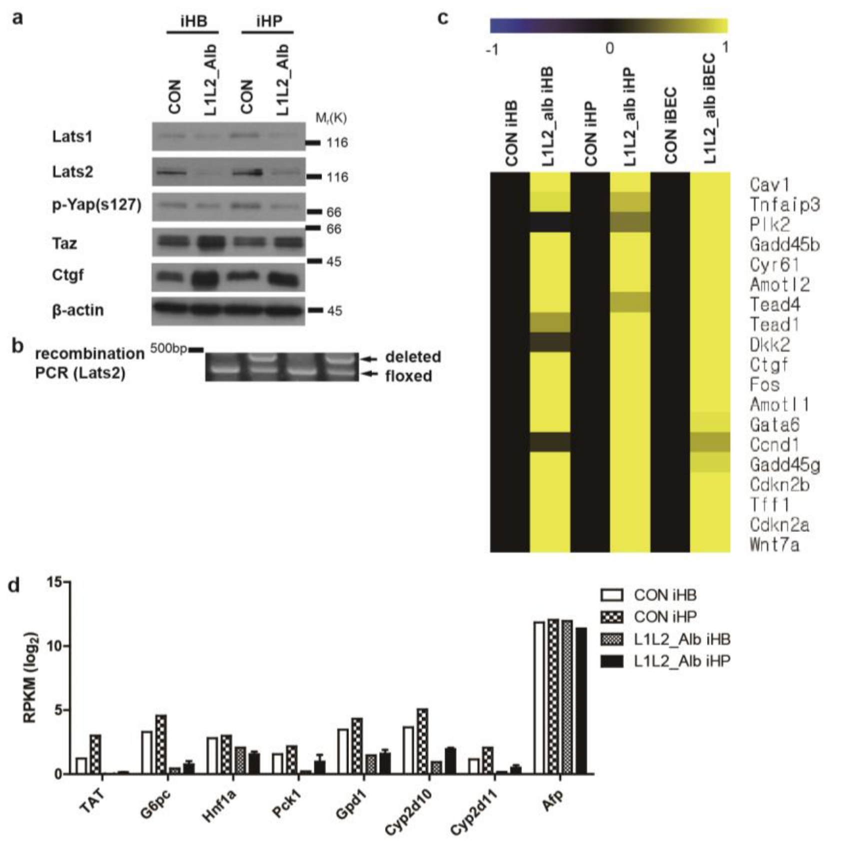

Supplementary Figure 1. Lats1/2 deleted iHBs and iHPs showed decreased transcripts of hepatocyte related genes (a and b) Western blots (a) and recombination PCR (b) of control and Lats1/2 deleted iHBs and iHPs (c) Heatmap for transcripts of Yap target genes from RNA-sequencing results with control and Lats1/2 deleted iHBs, iHPs and iBECs. (d) mRNA expression levels of hepatocyte-related genes from the RNA sequencing data with control and Lats1/2 deleted iHBs and iHPs. The data are presented as means ± SEM.

Supplementary Figure 2

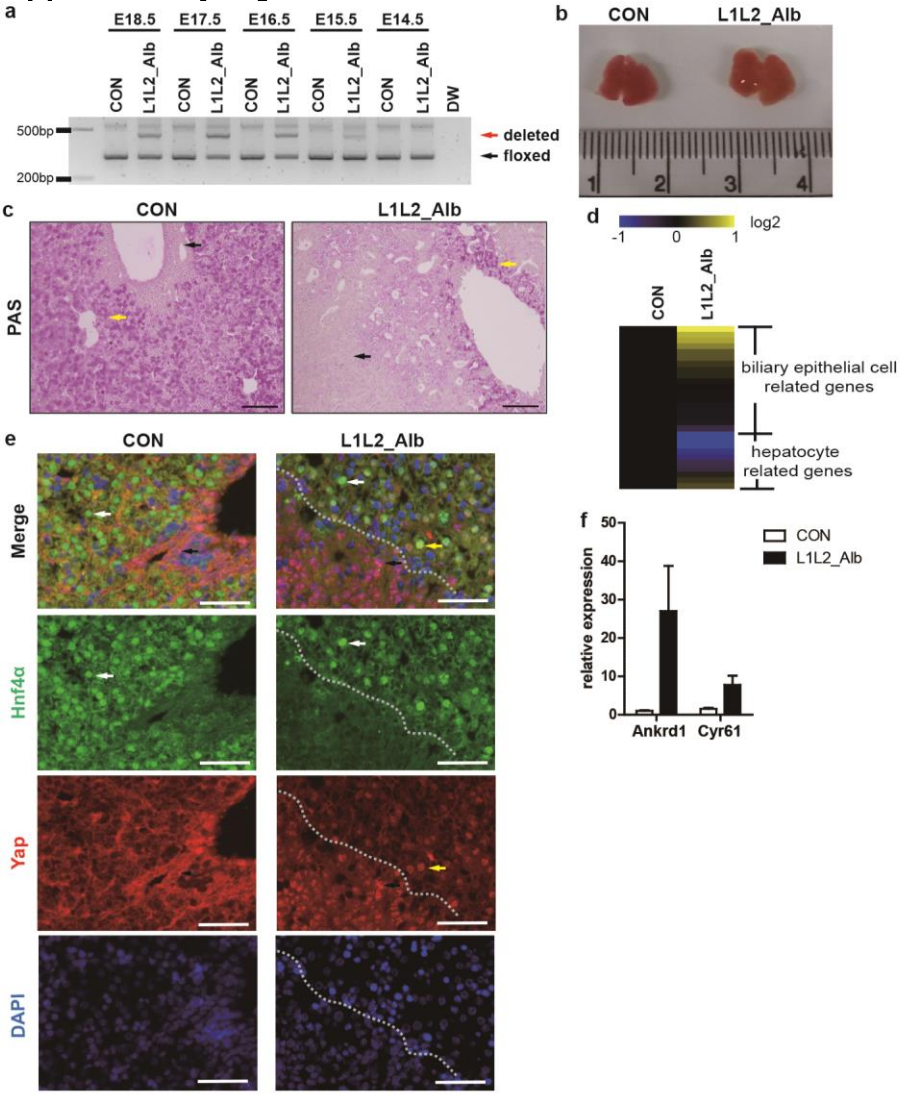

Supplementary Figure 2. Loss of Lats1/2 enhances immature biliary epithelial expansion by activating Yap and Taz (a) Recombination PCR of control and Lats1/2 deleted livers at each indicated embryonic day. (b) A photograph of postnatal day (P) 1 livers of the indicated genotypes. (c) PAS staining for detection of mature hepatocytes in control and L1L2_alb livers obtained at P1. Scale bars indicate 50 μm. (d) Heat map for BEC- and hepatocyte-related genes, as assessed by microarray analysis of mRNAs from control and L1L2_Alb livers sampled at P1. (e) Representative images obtained by immunofluorescence with antibodies against Yap and HNF4α in control and L1L2_alb livers sampled at P1. The white arrow indicates HNF4α-positive signals, and the yellow arrow indicates Yap and HNF4a double-positive signals. Scale bars indicate 100 μm. (f) Relative mRNA expression levels of the typical Yap target genes, Ankrd1 and Cyr61, in embryonic day (E) 17.5 whole livers, as assessed by qRT-PCR. The data are presented as means ± SEM.

Supplementary Figure 3

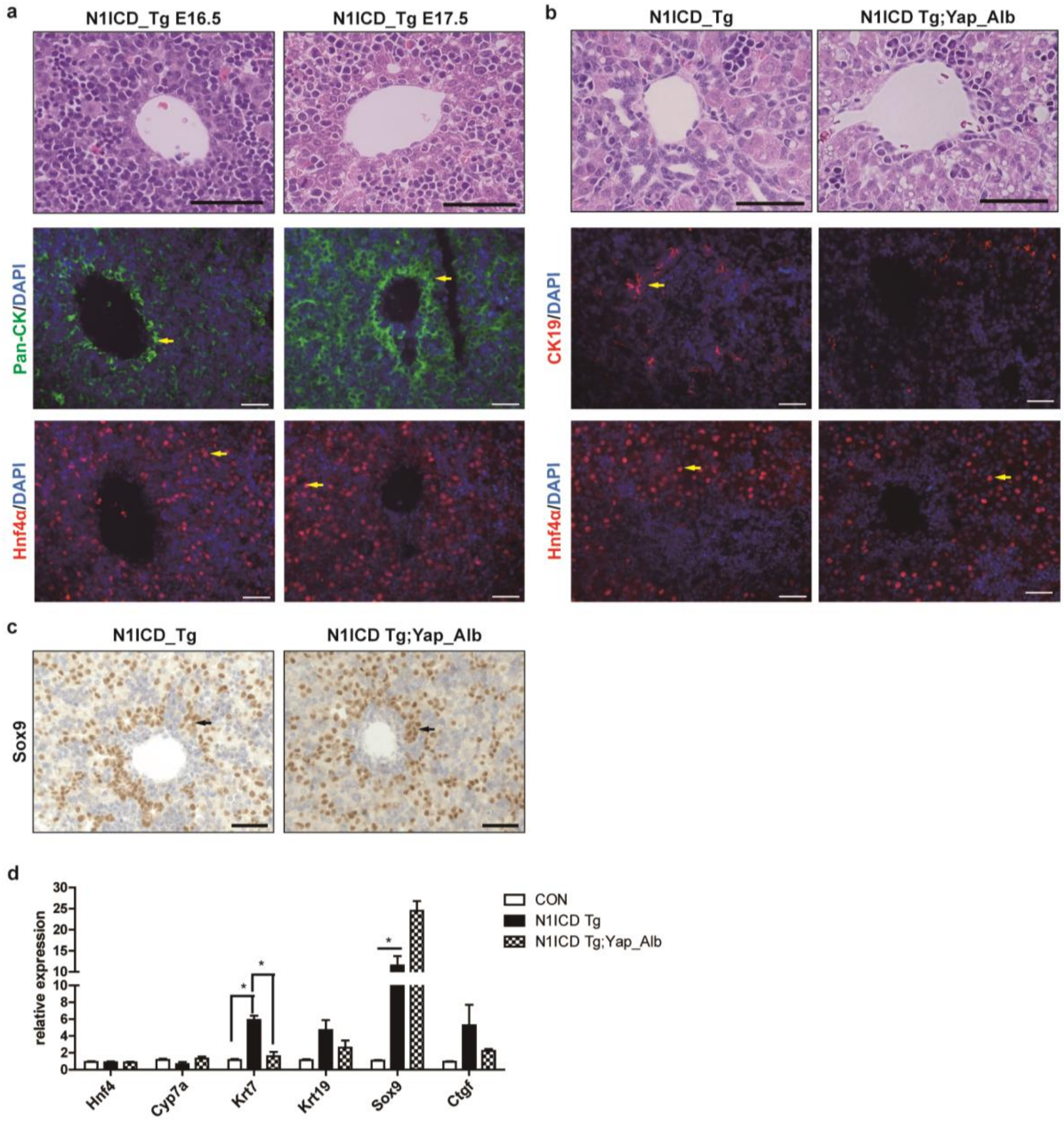

Supplementary Figure 3. Deficiency of Yap reduced biliary epithelial cell expansion by N1ICD over-expression (a) Representative H&E and immunofluorescence staining with anti-pan-CK and DAPI, and anti-Hnf4a and DAPI of N1ICD Tg livers at each indicated embryonic day. Yellow arrow indicate positive to anti-Pan-CK or anti-Hnf4a. (b) Representative H&E and immunofluorescence staining with anti-CK19 and DAPI, and anti-Hnf4a and DAPI of N1ICD Tg and N1ICD Tg;YAP_Alb livers at postnatal day 1 (P1). Yellow arrows indicate positive to anti-CK19 or anti-Hnf4α. (c) Representative immunohistochemistry staining with anti-Sox9 of N1ICD Tg and N1ICD Tg;Yap_Alb livers at P1. Black arrows indicate positive to anti-Sox9. (d) Relative mRNA expression levels of indicated genes in control, Yap_Alb, N1ICD Tg_Alb and N1ICD Tg;Yap_Alb livers at P1.
Scale bars indicate 50 μm; the data are presented as means ± SEM; *n=3*, *p < 0.05 (Student's *t*-test).

Supplementary Figure 4

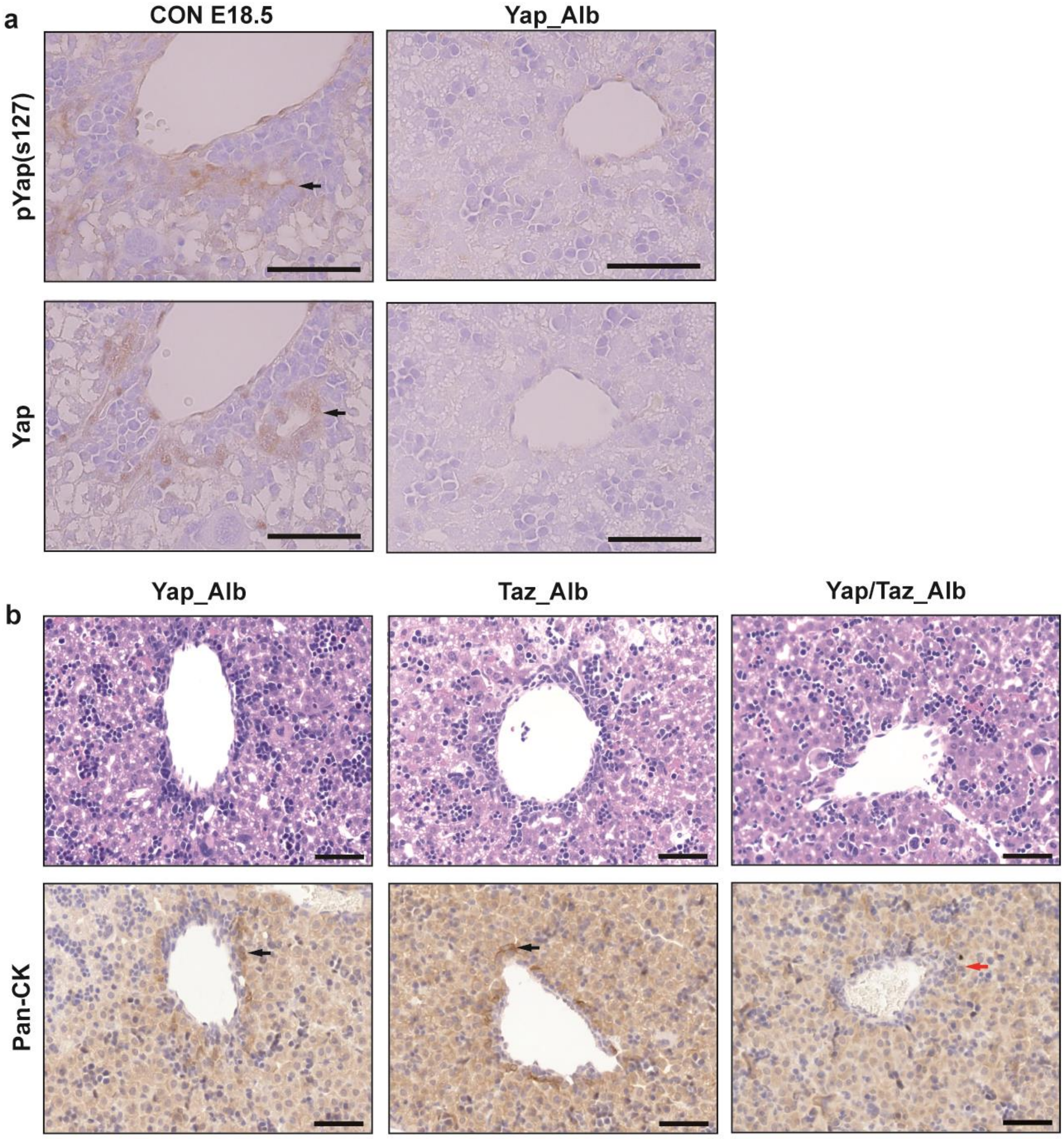

Supplementary Figure 4. Yap is activated during ductal plate formation (a) Representative immunohistochemistry performed with anti-pYap (S127) and anti-Yap in control livers at E18.5 and P1 Yap-knockout mice. Black arrows indicate positive signals to pYap or Yap. (b) Representative images of H&E and anti-panCK staining in livers from mice of the indicated genotypes. Black arrows indicate positive signals to pan-CK and red arrow indicates negative signal to pan-CK.

Scale bars indicate 50 μm.

Supplementary Figure 5

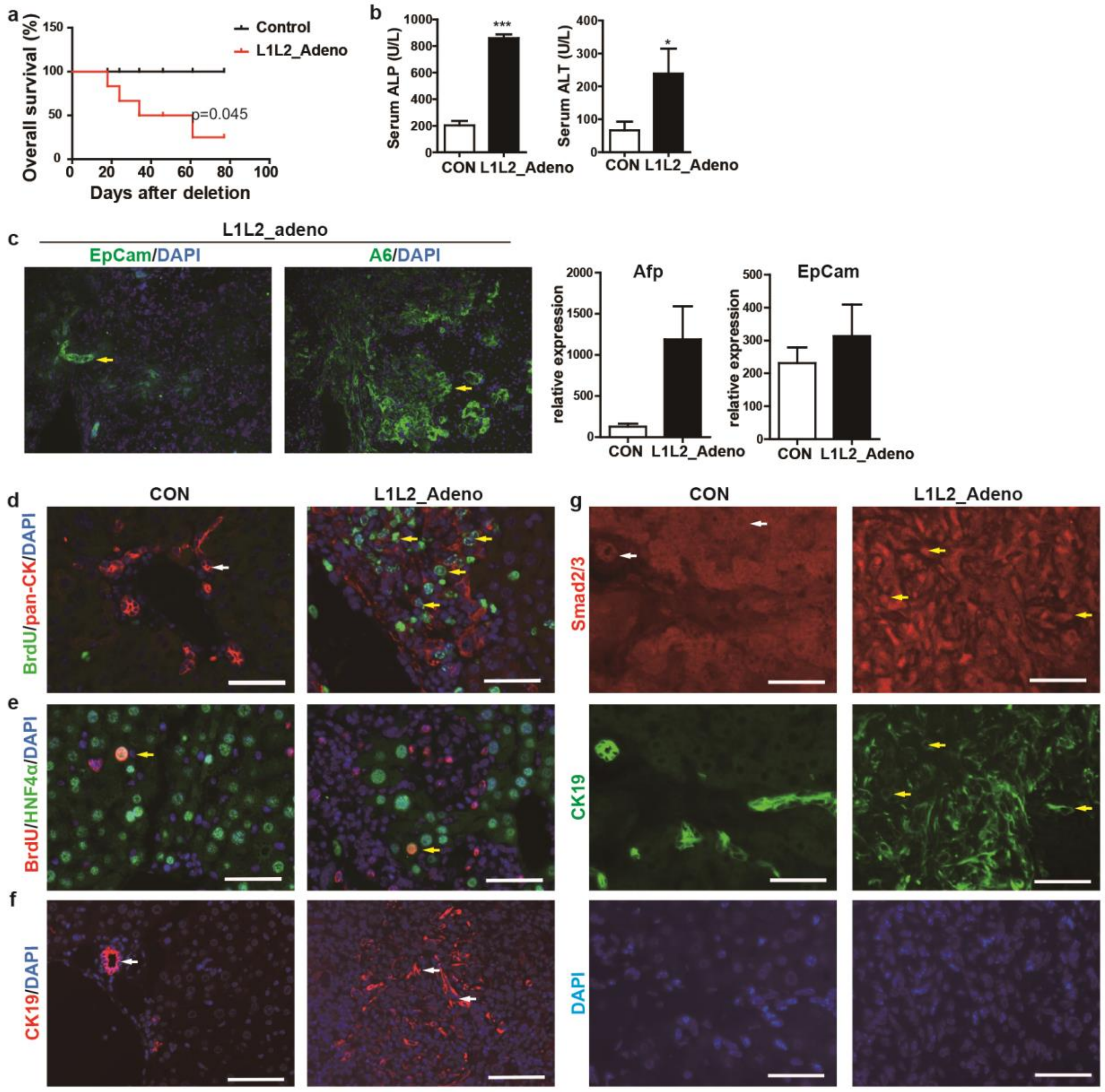

Supplementary Figure 5. Deletion of Lats1/2 in adult liver triggers the expansion of biliary epithelial cells (BECs) and fibroblasts (a) Survival curves for control and Lats1$^{-/-}$;Lats2$^{fl/fl}$ adeno-Cre injected mice (L1L2_Adeno) (2X10^{8} PFU; n = 6 mice per group; Log-rank test). (b) Serum test against alkaline phosphatase (ALP) and alanine aminotransferase (ALT) in control and L1L2_Adeno mice blood (*n=3*) (c) Expanded biliary cells expressed mainly A6 which is an oval cell reaction marker plus some Epcam which is a progenitor marker (left), whereas mRNA level of Afp, but not Epcam, was highly expressed in Lats-deficient livers (right). Yellow arrows indicate positive signals to EpCam or A6. (d and e) Representative immunofluorescence images obtained with anti-BrdU and anti-panCK (d) or anti-Hnf4α (e). Yellow arrows indicate double-positive cells, and white arrows indicate cells positive for BrdU alone. Scale bars indicate 100 μm. (f) Representative immunofluorescence images obtained with anti-CK19. White arrows indicate positive to anti-CK19. Scale bars indicate 100 μm (g) Single channel images of immunofluorescence staining results against anti-CK19, anti-Smad2/3 and DAPI (related to Fig. 5f). White arrows indicate cytoplasmic staining of Smad2/3 and yellow arrows indicate nuclear staining of Smad2/3. Scale bars indicate 50 μm.

The data are presented as means ± SEM; *n=3*, *p < 0.05, ***p < 0.001(Student's *t*-test).

Supplementary Figure 6

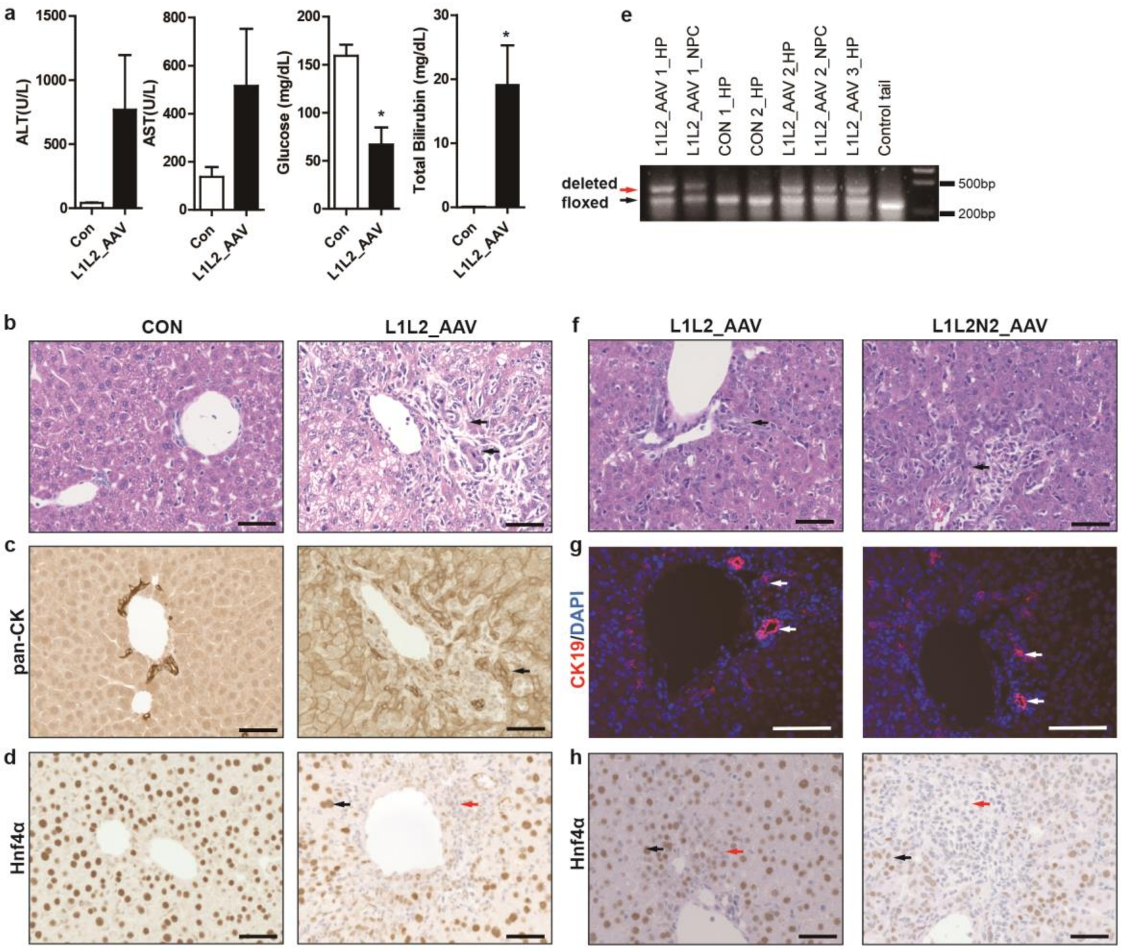

Supplementary Figure 6. Hepatocyte-specific deletion of Lats1/2 using the AAV-TBG-cre virus also induces biliary cell transition (a) Serum test against alanine aminotransferase (ALT), aspartate aminotransferase (AST), glucose and total bilirubin in control and L1L2_AAV mice blood (n=3). (b-d) Representative images of H&E staining (b) and immuohistochemical staining obtained with anti-panCK (c) and anti-Hnf4α (d) in control and L1L2_AAV livers. Black arrows in (b and c) indicate immature biliary epithelial cells. Black arrow in (d) indicates positive signal to anti-Hnf4a and red arrow indicates negative signal to anti-Hnf4a. Scale bars in (b-d) indicate 50 μm (e) Recombination PCR of Lats2 from control and L1L2_AAV hepatocytes or non-parenchymal cell fractions. (f-h) Representative images of staining H&E (f) and immunostaining obtained with anti-CK19 (g) and anti-Hnf4α (h) in L1L2_AAV and L1L2N2_AAV livers. Scale bars in (e) and (h) indicate 50 μm and scale bars in (g) indicate 100 μm.

Scale bars indicate 50 μm; the data are presented as means ± SEM; *n=3*, *p < 0.05 (Student's *t*-test).

Supplementary Figure 7

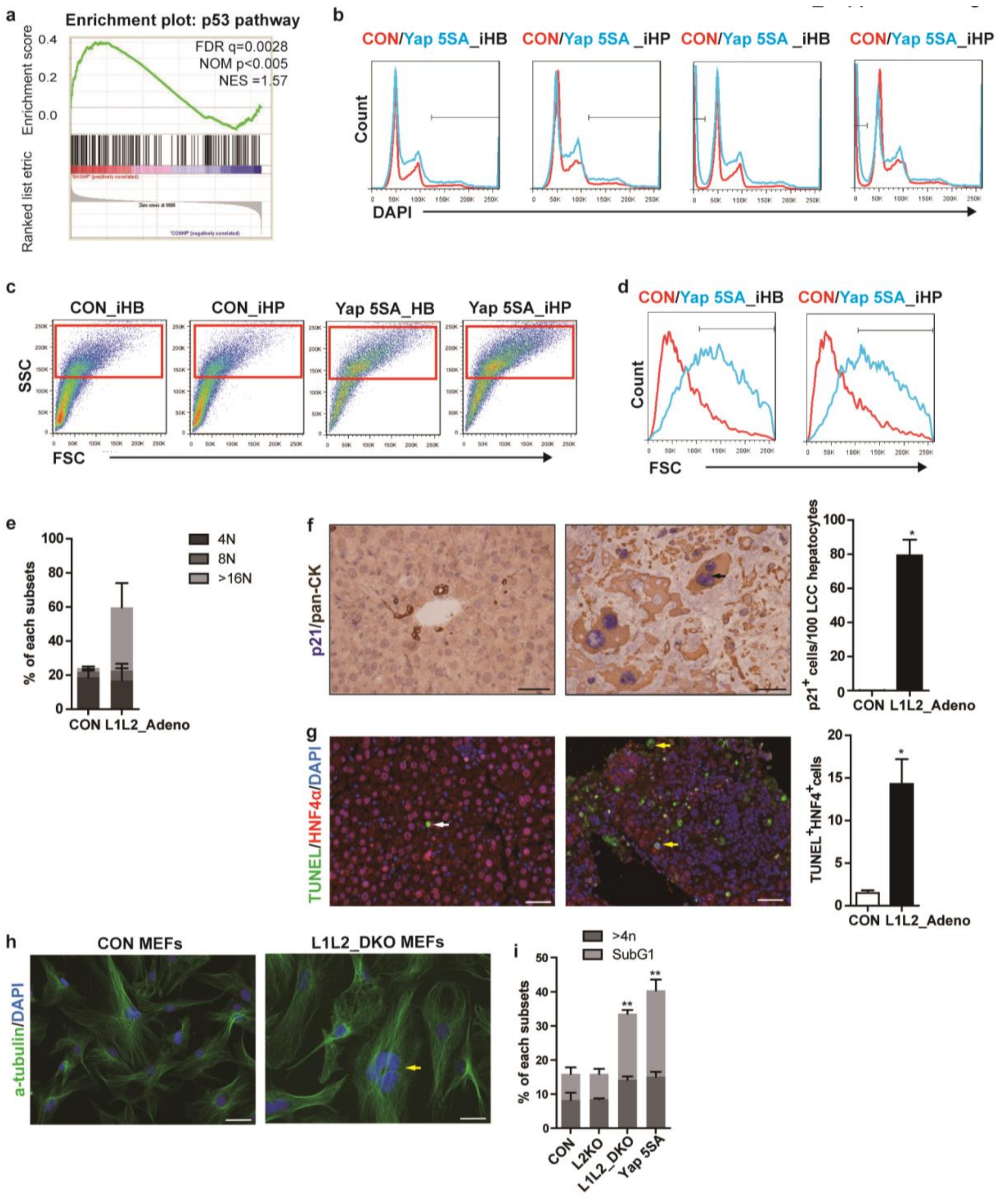

Supplementary Figure 7. Activated Yap-expressing hepatocytes or MEFs show enlargement, polyploidy and p53 pathway activation (a) Representative GSEA plot of p53 pathway genes that show up-regulation between control and Lats1/2-deficient iHPs. (b) Representative histogram shows >4n gating (first and second) and subG1 gating (third and fourth). (c) Representative FACS plots of live singlets and red rectangles indicate populations that were increased by over-expression of YAP 5SA. (d) Representative histogram about cell size of >4n gated cells. (e) Bar graphs of DNA contents, as measured by FACS of control and L1L2_Adeno livers. (f) Representative immunostaining images obtained with anti-p21 and anti-panCK in control and L1L2_Adeno livers and their quantifications. Scale bars indicate 50 μm (g) TUNEL assay performed by immunofluorescence staining with antibodies against HNF4α and their quantification. The white arrow indicates TUNEL-positive signals, while the yellow arrow indicates Yap and TUNEL double-positive signals. Scale bars indicate 100 μm. (h and i) Representative immunofluorescence staining with anti-tubulin (h) and DNA content analysis (i) of Lats1 and -2 knockout MEFs. Scale bars indicate 100 μm.

The data are presented as means ± SEM; *n=3,* *p < 0.05 and **p < 0.01 (Student's *t*-test).

Supplementary Figure 8

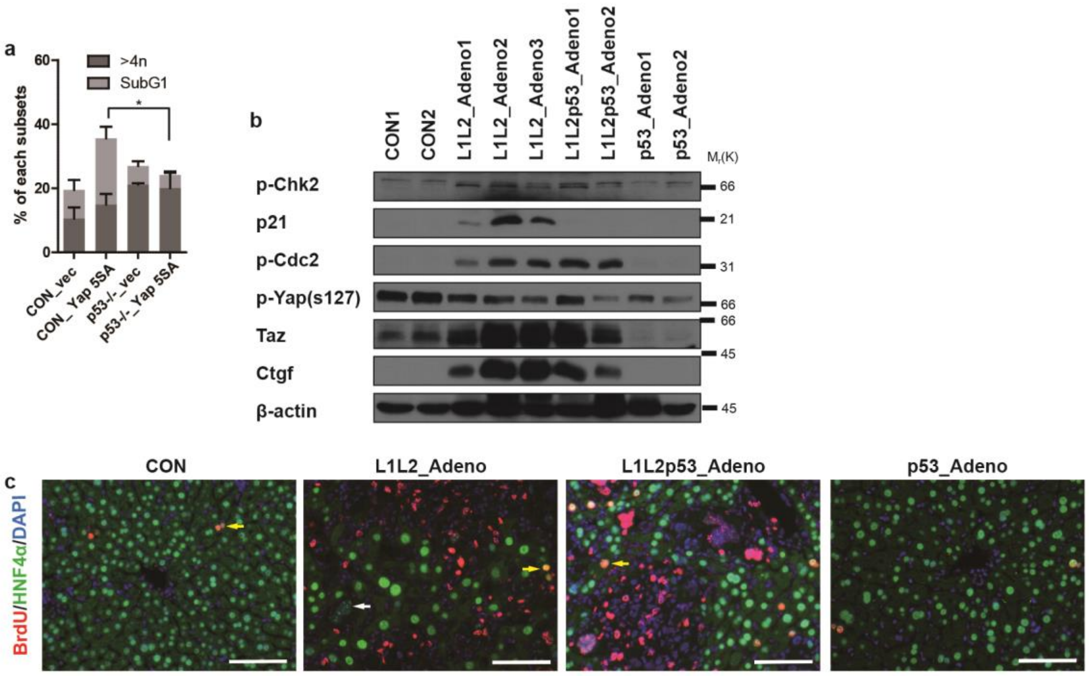

Supplementary Figure 8. Depletion of p53 rescues Yap-dysregulation-induced apoptosis and senescence in MEFs and livers (a) Bar graphs representing polyploidy and apoptosis in YAP 5SA over-expressing, control and $p53^{-/-}$ MEFs, as assessed by FACS. (b) Western blot analysis of protein extracts from control, L1L2_Adeno, L1L2p53_Adeno and p53_Adeno livers. (c) BrdU incorporation assay of livers from mice of the indicated genotypes. Yellow arrows indicate Hnf4a and BrdU double-positive signals.

Scale bars indicate 100 μm. The data are presented as means ± SEM; *n=3*, *p < 0.05 (Student's *t*-test).

Supplementary Figure 9

Figure 3b

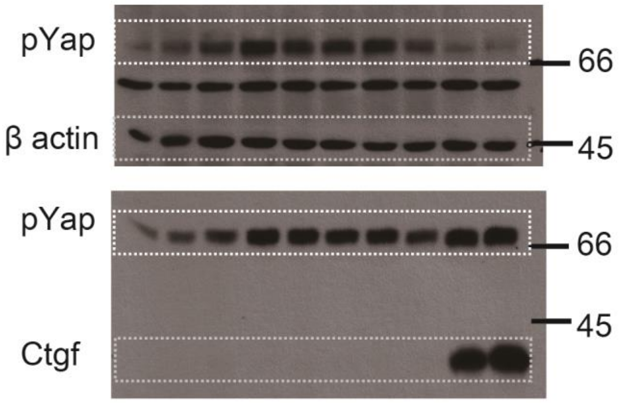

Supplementary Figure 1a

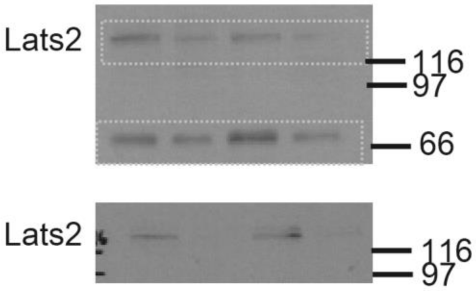

Supplementary Figure 8b

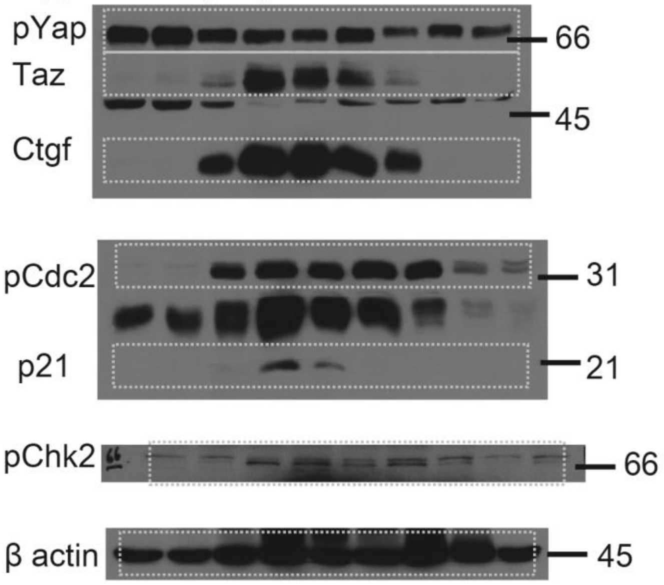

Supplementary Table 1

Gene Lists of Heat map in Supplementary Figure2d

Biliary epithelial cell markers

	CON P1	L1L2_Alb P1
KRT7	0.001	1.040451
KRT19	0.001	0.783539
JAG1	0.001	0.720546
FOXA2	0.001	0.562541
TGFB2	0.001	0.373114
ONECUT2	0.001	0.358512
OPN3	0.001	0.219247
TGFB3	0.001	0.138849
HNF1B	0.001	0.136638
HES1	0.001	0.035092
SALL4	0.001	-0.01428
TGFB1	0.001	-0.01521
NOTCH2	0.001	-0.0163
HEXB	0.001	-0.07661
HEXA	0.001	-0.0859
TBX3	0.001	-0.08723
DLK1	0.001	-0.13988
FOXA3	0.001	-0.41788

Hepatocyte markers

	CON P1	L1L2_Alb P1
CYP7A1	0.0001	-3.9248885
G6PC	0.0001	-1.7460995
CPS1	0.0001	-1.3435427
HNF4A	0.0001	-0.7895411
ALB	0.0001	-0.6913785
STAT3	0.0001	-0.4943089
CEBPA	0.0001	-0.4286007
HGF	0.0001	0.1039662
EGFR	0.0001	0.2025432
OSMR	0.0001	0.3166884

Average fold change expression of gene in L1L2_Alb P1 compared to expression of the gene in CON P1.

Supplementary Table 2

Gene Lists of Heat map in Figure 3c

	CON	L1L2_Alb
CTGF	0.0001	4.41817
ANKRD1	0.0001	3.64433
CYR61	0.0001	3.019909
BICC1	0.0001	2.430658
DAB2	0.0001	2.327472
PMP22	0.0001	1.714854
SCHIP1	0.0001	1.672068
DUSP1	0.0001	1.557649
GAS6	0.0001	1.482333
GADD45B	0.0001	1.284985
SGK1	0.0001	1.243097
TGFB2	0.0001	1.239459
CRIM1	0.0001	1.148064
AMOTL2	0.0001	1.099137
TSPAN3	0.0001	1.05384
MARCKS	0.0001	0.993043
FSCN1	0.0001	0.858903
PDLIM2	0.0001	0.801242
EMP2	0.0001	0.786937
PHGDH	0.0001	0.732843
LHFP	0.0001	0.720172
TGM2	0.0001	0.620576
FLNA	0.0001	0.452436
DDAH1	0.0001	0.395361
STMN1	0.0001	0.392695
MDFIC	0.0001	0.315468
SDPR	0.0001	0.161111
CDC20	0.0001	0.133092
ETV5	0.0001	0.116579
AXL	0.0001	0.089897
SHCBP1	0.0001	0.045979
ECT2	0.0001	-0.01239
BIRC5	0.0001	-0.04374
THBS1	0.0001	-0.04451
DUT	0.0001	-0.09466
FSTL1	0.0001	-0.15144
ITGB2	0.0001	-0.20685
NDRG1	0.0001	-0.24775
HEXB	0.0001	-0.25448

TOP2A	0.0001	-0.28779
TK1	0.0001	-0.3052
ITGB5	0.0001	-0.77714

Average fold change expression of gene in L1L2_Alb P1 compared to expression of the gene in CON P1.

Supplementary Table 3

qRT-PCR primers for mRNA expression

gene	species	Forward	Reverse
Krt7	mouse	GAC CCT TCA CGA GAC AGA GTT	GCA ATC TCA TTC CGG GTG TTG
Krt19	mouse	GGG GGT TCA GTA CGC ATT GG	GAG GAC GAG GTC ACG AAG C
Hnf1b	mouse	GAA AGC AAC GGG AGA TCC TC	CCT CCA CTA AGG CCT CCC TC
Hnf6	mouse	AGC CCT GGA GCA AAC TCA AGT CG	TGC ATG TAG AGT TCG ACG TTG GAC
Notch2	mouse	ATG CAC CAT GAC ATC GTT CG	GAT AGA GTC ACT GAG CTC TCG
Hey1	mouse	AGA AGG CTG GTA CCC AGT GCC TT	CAA ACT CCG ATA GTC CAT AGC CA
Sox9	mouse	GAG CCG GAT CTG AAG AAG GA	GCT TGA CGT GTG GCT TGT TC
Hes1	mouse	CCA GCC AGT GTC AAC ACG A	AAT GCC GGG AGC TAT CTT TCT
Hnf4a	mouse	CAC GCG GAG GTC AAG CTA C	CCC AGA GAT GGG AGA GGT GAT
Cyp7a1	mouse	AGC AAC TAA ACA ACC TGC CAG TAC TA	GTC CGG ATA TTC AAG GAT GCA
Albumin	mouse	CGA GAA GCT TGG AGA ATA TGG	GTC AGA GCA GAG AAG CAT GG
Cebpa	mouse	GAA CAG CAA CGA GTA CCG GGT A	CCC ATG GCC TTG ACC AAG GAG
Ankrd1	mouse	CGA CTC TTG ATG ACC TTC GG	ATT GCT TTG GTT CCA CTC TG
Cyr61	mouse	CCC CCG GCT GGT GAA AGT	GCG GTT CGG TGC CAA AGA
Ctgf	mouse	GGG CCT CTT CTG CGA TTT C	ATC CAG GCA AGT GCA TTG GTA
Tgfb2	mouse	TGG AGT TCA GAC ACT CAA CAC A	AAG CTT CGG GAT TTA TGG TGT
Vimentin	mouse	CGG CTG CGA GAG AAA TTG C	CCA CTT TCC GTT CAA GGT CAA G
β-actin	mouse	GAA ATC GTG CGT GAC ATC AAA G	TGT AGT TTC ATG GAT GCC ACA G
Collagen I	mouse	GCT CCT CTT AGG GGC CAC T	CCA CGT CTC ACC ATT GGG G

qRT-PCR primers for Chromatin immunoprecipitation analysis

Tgfb2 R1	mouse	GAG ATT TGG AGG AGC AGG GCT G	CGG CGA CTT TCC CAT GAC TTC
Tgfb2 R2	mouse	TGC TGG CCT AAT GAA GTG GAA	GGT CCT GAG TTC AAG TCC CAG
Tgfb2 R3	mouse	TGA AAC CTC AAG TCC ACC CCC C	GCT CTA AAC ACC ACA GCC CTC

Hnf4a R1	mouse	ACC CTG TGC TGC CAG TTT CTC	CCT GGT CCC AAG GTT ATC CTT
Hnf4a R2	mouse	AAG GCT GAG GCA GGA GTG TCA	CTC ACC CCC AAA CCA TCA TTT
Hnf4a R3	mouse	GGC CCT ATC TTC GGG CCC CTG	GTG CCT CCT GTC ACA ATC ACT
Pou5f1	mouse	GGC AGA CGG CAG ATG CAT AAC	CTC AAT AGC AGA TTA AGG AAG GGC
Ctgf	mouse	CAA TCC GGT GTG AGT TGA TG	GGC GCT GGC TTT TAT ACG
